# Phytochemical characterization and correlation analysis of nutritional value in sweet pepper (*Capsicum annuum* L.) genotypes at various growth stages

**DOI:** 10.3389/fpls.2025.1719537

**Published:** 2026-02-11

**Authors:** Hemlata Bharti, Nandimandalam Hemalatha, Rukhsana Malita, Vinod Kumar Sharma, Rakesh Bharadwaj, Prashanth Babu

**Affiliations:** 1 Centre for Protected Cultivation Technology, ICAR-Indian Agricultural Research Institute, New Delhi, India; 2 ICAR-National Bureau of Plant Genetic Resources, New Delhi, India; 3 Division of Genetics, ICAR-Indian Agricultural Research Institute, New Delhi, India

**Keywords:** sweet pepper, fruit pigmentation, bioactive compounds, maturity stage, metabolite profiling

## Abstract

Sweet pepper (Capsicum annuum) is valued globally for its nutritional richness and vibrant colours, with significant commercial and therapeutic potential. This study analysed 49 sweet pepper genotypes (green, red, orange, white, purple, chocolate, yellow) at unripe and mature stages to assess bioactive compounds: vitamin C, total phenols, total soluble sugars, chlorophylls, anthocyanins, carotenoids, and xanthophylls. Results showed considerable variation in vitamin C, ranging from 343.00 (immature) to 12,565.95 µg/g in mature fruits, with promising genotypes CPCT-31A-5 and NBR-22. Antioxidant levels increased from 7.32 (immature) to 431.34 µg/g at maturity, notably in AVR-152, CPCT-32C, and 33B. Total phenols ranged from 146.0 to 6,707.50 µg/g, peaking in mature fruits, especially in AVR-27, NBR-20, F5-32C-3, and Chocolate. Carotenoids and xanthophyll also increased at maturity, with AVR-141 showing the highest content (8.77 µg/g). White capsicum exhibited the highest total soluble solids (43,730.5 µg/g) at the immature stage, while mature orange-red fruits had the richest vitamin C (12,565.95 µg/g) and antioxidants. NBR-10 stood out for chlorophyll content, suggesting potential for photosynthetic studies. Purple capsicum showed the highest anthocyanin content (15.50 µg/g) at the immature stage. Strong correlations were observed between vitamin C and phenols (r = 0.79), antioxidants and vitamin C (r = 0.80), and antioxidants and phenols (r = 0.96) at the immature stage. These findings highlight the dynamic interplay of bioactive compounds across genotypes and maturity stages, with implications for breeding and dietary applications. The study underscores the significant variation in bioactive compounds across sweet pepper genotypes and maturity stages, emphasizing the potential of specific genotypes like CPCT-31A-5, AVR-27, and NBR-10 for enhanced nutritional and breeding outcomes. These insights can guide future research on fruit quality improvement and functional food development.

## 1 Introduction

Sweet pepper, the non-pungent form of *Capsicum annuum* (2n = 24), is a key functional food owing to its rich biochemical profile. The genus *Capsicum* comprises 42 species, of which five—*C. annuum, C. frutescens, C. chinense, C. baccatum*, and *C. pubescens*—are fully domesticated and widely cultivated, forming an essential component of global cuisines and agricultural systems. The remaining species, though semi-domesticated or wild, contribute valuable genetic diversity for breeding and conservation (Pickersgill, 1997). Originating in the tropical and subtropical regions of the Americas, Mexico represents the principal centre of diversity for *Capsicum* (Bosland and Votava, 2012). Fruit colouration in capsicum spans green, yellow, orange, red, purple, and even black, resulting from chlorophyll degradation and the progressive accumulation of carotenoids and anthocyanins (Ha et al., 2007; Liu et al., 2018; Lightbourn et al., 2008). Carotenoids such as β-carotene and capsanthin act as potent antioxidants and vitamin A precursors, while anthocyanins contribute additional antioxidant and stress-mitigating functions (Deli et al., 2001; Matsufuji et al., 2007). Over the past two decades, global consumption of coloured peppers has risen significantly due to their sensory qualities and recognized health benefits (Mongkolporn and Taylor, 2011; Baenas et al., 2019).

Fresh peppers are especially rich in bioactive compounds; red and yellow fruits contain 400–500 mg/100 g (dry weight) vitamin C and 350–700 mg/100 g (dry weight) β-carotene (Tchiegang et al., 1999). Vitamin C (ascorbic acid, AsA)—present as ascorbic acid (AA) and its oxidized form dehydroascorbic acid (DHA)—is vital for human health due to its strong antioxidant activity (Palma et al., 2011). It prevents LDL cholesterol oxidation, a precursor to arterial plaque formation (Howard et al., 2000; Marín et al., 2004; Perucka and Materska, 2007; Araceli et al., 2007; Zaki et al., 2013), while deficiency leads to scurvy and heightened risks of cardiovascular diseases, cataracts, cancer, and age-related disorders (Xu et al., 2001). In plants, AsA plays a critical role in mitigating oxidative stress caused by drought, salinity, freezing, and pathogen attack, as shown in crops such as potato and tomato (Martí et al., 2009; Ahmad et al., 2014; Min et al., 2020).

Antioxidant means “against oxidation.” Antioxidants work to protect lipids from peroxidation by radicals. They inhibit or delay the oxidation of other molecules by inhibiting the initiation or propagation of oxidizing chain reactions. Antioxidants are effective because they are willing to give up their electrons to free radicals. When a free radical gain the electron from an antioxidant it no longer needs to attack the cell and the chain reaction of oxidation is broken (Dekkers et al., 1996). There are two basic categories of antioxidants, namely, synthetic, and natural. In general, synthetic antioxidants are compounds with phenolic structures of various degrees of alkyl substitution, whereas natural antioxidants of plant regions are classified as vitamins, phenolic compounds, or flavonoids (El-Ghorab et al., 2013). The high popularity of sweet peppers is closely linked to their antioxidant richness. Total antioxidant activity results from multiple bioactive constituents, particularly flavonoids, which influence nutritional quality (Dobón-Suárez et al., 2021; Sanatombi, 2023). These compounds neutralize free radicals and inhibit oxidative chain reactions (El-Ghorab et al., 2013; Kurucz et al., 2023). *Capsicum* ranks among the top vegetables for phenolic concentration, surpassing nutrient-dense crops such as spinach, broccoli, and garlic (Singh, 2024). Phenolic content ranges between 0.058 and 0.085 mg/100 g depending on cultivar and environmental conditions (Perucka and Materska, 2007). Polyphenols—comprising phenolic acids, flavonoids, lignans, and stilbenes—are central to flavour, colour, bitterness, and pungency, with levels varying during fruit maturation (Estrada et al., 2000; Kamal et al., 2017; Lo’ay and EL-Khateeb, 2018; Saleem et al., 2021). Owing to their antioxidant potential, they are recognized as important dietary supplements (Steelheart et al., 2020). In addition to phenolics, sweet peppers are rich in provitamin A carotenoids (β-carotene) and xanthophylls, essential for human nutrition and for imparting vivid fruit colouration (Deli et al., 2001; Wall et al., 2001; Wahyuni et al., 2011). β-Carotene, synthesized in green, yellow, and orange tissues, contributes directly to the total carotenoid pool and serves as a precursor of orange and red pigments in mature fruits (Guzman et al., 2010). The structural diversity of carotenoids not only enhances antioxidant defence and visual health benefits but also determines marketability through vibrant pigmentation. Anthocyanins and chlorophylls further contribute to capsicum’s functional and aesthetic qualities. Anthocyanins—derived from pelargonidin, cyanidin, delphinidin, peonidin, petunidin, and malvidin—support pollination, seed dispersal, and protection from ultraviolet radiation, herbivores, and pathogens (Liu et al., 2018; Chalker-Scott, 1999; Kong et al., 2003; Pojer et al., 2013). Chlorophylls a and b are essential for photosynthesis and dominate immature green fruits; their degradation during ripening enables carotenoid accumulation and colour transition. Chlorophyll also has commercial applications as a natural pigment in food and cosmetics (Riga et al., 2005; Pandey et al., 2021). Despite extensive global consumption, a comprehensive understanding of capsicum’s full spectrum of bioactive compounds, pigment biosynthesis pathways, and stress-response mechanisms remains incomplete. Research has largely focused on limited genotypes, leaving substantial gaps regarding the nutritional and functional diversity across the wide capsicum gene pool. The present study addresses this gap by analysing 49 diverse *Capsicum* genotypes, evaluating their bioactive compound profiles across different colours—red, yellow, orange, white, purple, and chocolate—at two maturity stages. This work provides the first consolidated analysis encompassing all major sweet pepper colour groups within a single investigation. By linking biochemical traits with fruit colour and maturity, the study identifies key compounds influencing antioxidant capacity and nutritional quality, determines which colour groups possess superior health attributes, and defines the optimal consumption stage for maximizing nutritional benefits. These insights offer valuable guidance for breeding programs aimed at improving nutritional value, functional properties, and stress resilience in sweet peppers.

## 2 Materials and methods

### 2.1 Plant material and experimental conditions

**Figure 1:**
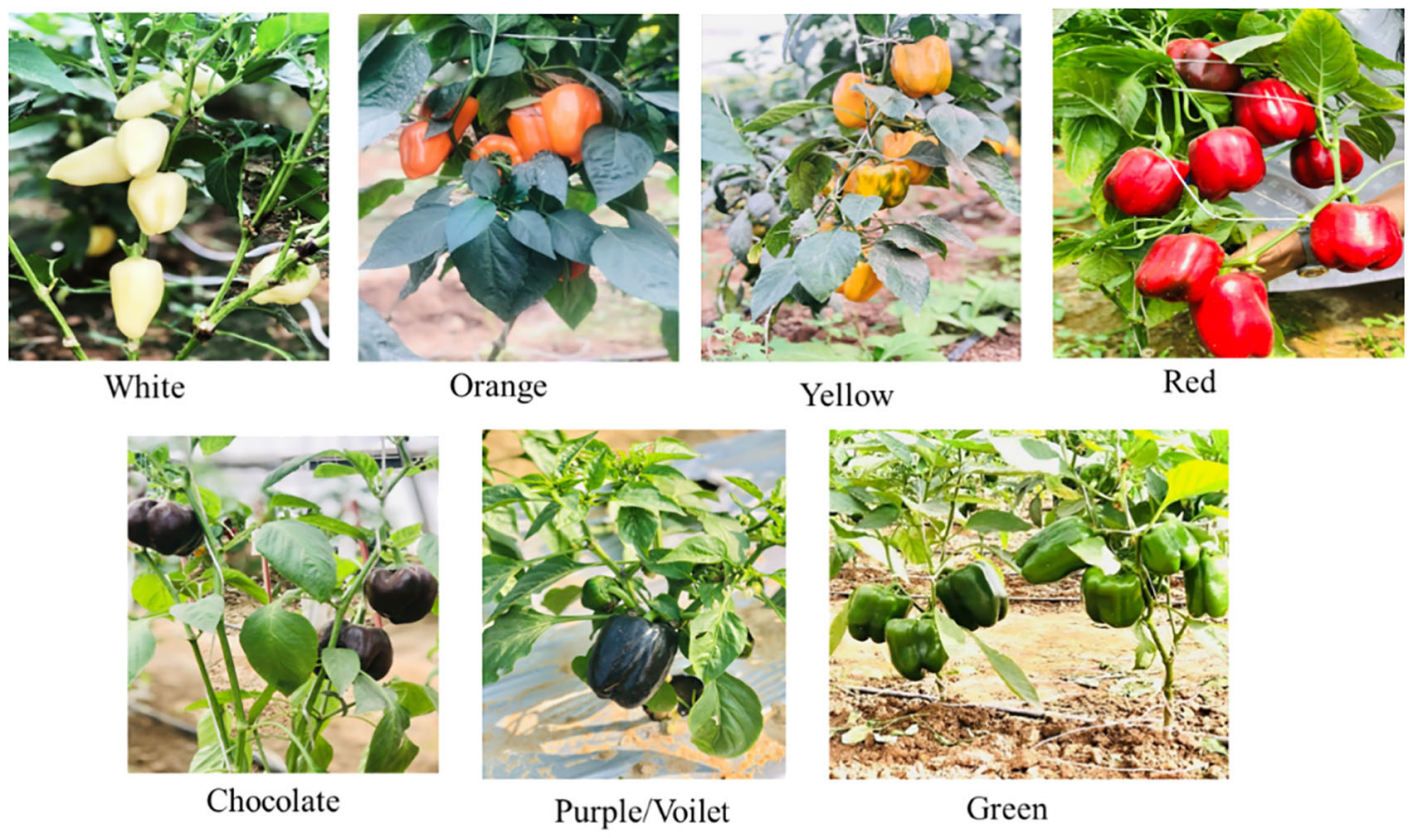
Genotypes of sweet peppers based on different colours as on ASTA colour chart (13- White, 80- Orange, 40- yellow, 100- Red, 150- Chocolate, 120- Purple and 20- Green) used in the present study.

A total of 49 sweet pepper (*Capsicum annuum*) genotypes were grown under a naturally ventilated polyhouse at the Centre for Protected Cultivation Technology (CPCT), ICAR-IARI, New Delhi (28°38’24.02” N, 77°10’26.33” E). All genotypes were cultivated during the main capsicum growing seasons of 2022–23 and 2024 in a randomized block design (RBD) with four replications. Transplanting was done at a spacing of 30 cm × 60 cm (row × plant), and crop management followed standard recommended practices to ensure optimal trait expression. Biochemical estimation of quality and bioactive components from different coloured capsicum fruits (green, white, orange, red, chocolate, purple, and orange-red) was conducted at the ICAR–National Bureau of Plant Genetic Resources (ICAR-NBPGR), New Delhi (**Figure 1**). As the study focused exclusively on biochemical and bioactive compound analyses, no morphological characterization or correlation analysis was performed. The fruit maturity stages were classified based on external colour development. The immature stage was defined by the retention of green, white, purple and chocolate colour (chlorophyll-dominant) and undeveloped seeds. The mature stage was defined by the characteristic ripe colour of the respective Capsicum type (red, orange, yellow) and developed seeds.

### 2.2 Phytochemical analysis and data collection

This study focused on the quality analysis of vitamin- C content, antioxidant, chlorophylls and carotenoids, anthocyanin analysis, total phenols and total soluble sugar (**Supplementary Table S1**).

#### 2.2.1 Estimation of vitamin C content

The vitamin C content in the samples was estimated using UV-spectrophotometric analysis (Mohammed et al., 2009). Initially, 1 g of each sample was weighed and ground in a mortar and pestle with 5 mL of a 4.5% m-phosphoric acid solution. The resulting turbid extract was transferred into a 10 mL centrifuge tube and centrifuged (SIM BIOCHEM Model-T-24BL) at 12,000 rpm for 15 minutes.

The clear supernatant was collected into a fresh centrifuge tube, and the residue was re-extracted with 5 mL of a 3% m-phosphoric acid solution. The mixture was centrifuged again at 12,000 rpm for 15 minutes. The supernatant from the second centrifugation was pooled with the previous supernatant to make up the total volume to 10 mL using the 3% m-phosphoric acid solution. For analysis, 1 mL of the extracted sample was pipetted into a 15 mL test tube and dissolved in 200 µl of 3% m-phosphoric acid solution. To this, 2.8 mL of distilled water and 400 µl of Folin & Ciocalteu’s reagent (diluted 1:4) were added. The contents of the test tube were mixed thoroughly by vortexing. After the second vortex mixing, the absorbance of the sample was measured at 650 nm using a reagent blank consisting of 1.2 mL of 3% m-phosphoric acid, 2.8 mL of distilled water, and 0.4 mL of Folin & Ciocalteu’s reagent (1:4). This process allowed the quantification of vitamin C based on the absorbance value. From the standard curve by plotting concentration of std. on ‘x’ axis versus absorbance on ‘y’ axis find out the concentration of vitamin-C in the test sample and express as µg/g.


$$\begin{array}{c} \text{\% Amount of vitamin C present in sample }=\\ \frac{\text{Vitamin C value from graph }\left(\text{µg}\right)\;\times \text{ Total volume of extract }\left(\text{mL }\right)\;\times \;100\;}{Aliquot\;Sample\;Used\;\left(ml\right)\times Weight\;of\;Sample\;\left(g\right)} \end{array}$$

#### 2.2.2 Estimation of antioxidant content

Antioxidant activity was determined using the FRAP (Ferric Reducing Antioxidant Power) assay following Benzie and Strain (1996) with minor modifications. One gram of sample was homogenized in 5 mL of 80% ethanol and centrifuged at 10,000 rpm for 10 minutes. The residue was re-extracted with 80% ethanol, centrifuged again and both supernatants were combined and adjusted to 10 mL. For analysis, 50 µL of extract was mixed with 250 µL distilled water and 2.2 mL FRAP reagent (acetate buffer: ferric chloride: TPTZ = 10:1:1). After 1 hour of reaction and vortexing, absorbance was recorded at 593 nm using a reagent blank. Antioxidant content was calculated from a gallic acid standard curve (concentration vs. absorbance) and expressed as µg/g, and later converted to percentage for plotting.


$$\begin{array}{c} \text{\% Antioxidant present in sample}=\\ \frac{\text{Antioxidant value from graph}\left(\text{µg}\right)\text{ x Total volume of extract }\left(\text{mL }\right)\text{ x }100}{Aliquot\;Sample\;Used\;\left(ml\right)\times Weight\;of\;Sample\;\left(g\right)} \end{array}$$

#### 2.2.3 Estimation of total phenols content

Total phenols were estimated using the Folin–Ciocalteu method (Singleton and Rossi, 1965). One gram of sample was homogenized in 5 mL of 80% ethanol and centrifuged at 10,000 rpm for 10 minutes. The residue was re-extracted, centrifuged again, and both supernatants were pooled and made up to 10 mL with 80% ethanol. For analysis, 100 µL of extract was evaporated to dryness in a boiling water bath. After cooling, 3 mL distilled water and 0.5 mL Folin–Ciocalteu reagent (1:1) were added and mixed. After 3 minutes, 2 mL of 20% Na_2_CO_3_ was added, and the mixture was incubated for 1 hour at room temperature. Absorbance was measured at 650 nm against a reagent blank. Total phenol content (µg/g) was calculated using a standard curve prepared from known phenolic standards (concentration vs. absorbance).


$$\begin{array}{c} \%\text{Phenols present in sample }=\\ \frac{\text{Phenol value from graph }\left(\text{µg}\right)\text{ x Total volume of extract }\left(\text{mL }\right)\text{ x }100}{\text{Aliquot Sample Used }\left(\text{mL }\right)\times \text{Weight of Sample }\left(\text{g}\right)} \end{array}$$

#### 2.2.4 Estimation of total soluble sugar

Total soluble sugars were estimated using the anthrone reagent method (Irigoyen, 1992). One gram of sample was homogenized in 5 mL of 80% ethanol and centrifuged at 10,000 rpm for 10 minutes. The residue was re-extracted, centrifuged again, and both supernatants were pooled and adjusted to 10 mL. For analysis, 100 µL of extract was evaporated to dryness in a boiling water bath. The residue was dissolved in 1 mL distilled water, followed by 4 mL chilled anthrone reagent, and the mixture was vortexed. The tubes were heated for 8 minutes in a boiling water bath, cooled, and absorbance was measured at 630 nm against a reagent blank. Total soluble sugar content (µg/g) was determined using a glucose standard curve (concentration vs. absorbance).


$$\begin{array}{c} \%\text{ Carbohydrates prese}nt\;in\;sampl\text{e}=\\ \frac{\text{Sugar Value }\left(\text{µg}\right)\text{ x Total volume of extract }\left(\text{mL }\right)\text{ x }100}{Aliquot\;Sample\;Used\;\left(ml\right)\times Weight\;of\;Sample\;\left(g\right)} \end{array}$$

#### 2.2.5 Estimation of anthocyanin content

The anthocyanin pigment content was estimated using the pH differential method (Lee et al., 2005). First, 1 g of each fresh sample was weighed, and 5 mL of 0.1N HCl was added. The mixture was then ground using a mortar and pestle. The turbid pigment extract was transferred to a 10 mL centrifuge tube and centrifuged at 10,000 rpm for 10 minutes, after which the clear supernatant was collected into a fresh centrifuge tube, bringing the total volume of the extract to 5 mL. Two dilution sets were prepared for each sample: in Set 1, 1 mL of the extracted sample was mixed with 3 mL of sodium acetate buffer (pH 4.5), and in Set 2, 1 mL of the extract was combined with 3 mL of HCl-KCl buffer (pH 1.0). The absorbance of each dilution was measured at both 520 nm and 700 nm using a spectrophotometer, with measurements taken against a blank that consisted of the respective buffer (sodium acetate for pH 4.5 or HCl-KCl for pH 1.0) and 1 mL of 0.1N HCl. This method takes advantage of the pH-dependent absorbance properties of anthocyanins, as the pigments exhibit different absorbance levels at acidic (pH 1.0) and less acidic (pH 4.5) conditions, allowing for an accurate estimation of the total anthocyanin content. Calculation of anthocyanin pigment concentration, expressed as cyanidin-3-glucoside equivalents, as follows:

Anthocyanin pigment (cyanidin-3-glucoside equivalents, mg/L) = A × MW × DF × 10^3^/ϵ × 1

Where, A = (*A*_520nm_ -*A*_700nm_) pH 1.0 - (*A*_520nm_ - *A*_700nm_) pH 4.5; MW (molecular weight) = 449.2 g/mol for cyanidin-3-glucoside (cyd-3-glu); DF = dilution factor; 1 = pathlength in cm; ϵ = 26900 molar coefficient, in L mol^-1^ cm^-1^, for cyd-3-glu; and 10^3^= factor for conversion from g to mg.

#### 2.2.6 Estimation of chlorophylls and carotenoids

Chlorophylls and carotenoids were estimated using UV-spectrophotometric analysis (Hornero-Méndez and Mínguez-Mosquera, 2001). A sample weighing between 30–50 mg of fresh tissue was prepared by adding 5 mL of 100% acetone (for ≤50 mg samples) and 100–200 mg of magnesium oxide (MgO) to neutralize plant acids and prevent the formation of pheophytin a. The mixture was ground using a mortar and pestle. The turbid pigment extract was then transferred to a 10 mL centrifuge tube and centrifuged at 10,000 rpm for 10 minutes. The clear supernatant was collected in a fresh centrifuge tube for analysis. Absorbance readings were taken using a 1-cm path length cuvette in a UV-VIS spectrophotometer (Cat#BT-VS-E:20190103002, India), with acetone as the solvent blank. The readings were taken at five specific wavelengths: 750 nm (to check for clarity of the extract, A750 = 0), 661.6 nm (chlorophyll a maximum), 644.8 nm (chlorophyll b maximum), 520 nm (to assess green plant/fruits tissue extract, A520 should be<10% of A661.6), and 470 nm (for carotenoids). The absorbance values obtained were then applied to the appropriate equations for each solvent system, allowing for the calculation of the pigment content (µg/g of extract solution). Pigment concentrations in µg/g extract solution are obtained by putting absorbance values at specific wavelengths for specific solvent systems.


$$\text{Chl}a\left(µ\text{g}/\text{g }\right)=\left({\text{C}}_{\text{a}}\right)$$

$$\text{Chl}b\left(µ\text{g}/\text{g }\right)=\left({\text{C}}_{\text{b}}\right)$$

$$\text{Carotenoids and Xanthophylls }\left(µ\text{g}/\text{g}\right)={\text{C}}_{\left(\text{x}+\text{c}\right)}$$

Acetone (pure Solvent)


$${\text{C}}_{\text{a}}=11.24\;{\text{A}}_{661.6}-2.04\;{\text{A}}_{644.8}$$

$${\text{C}}_{\text{b}}=20.13\;{\text{A}}_{644.8}-4.19\;{\text{A}}_{661.6}$$

$${\text{C}}_{\left(\text{x}+\text{c}\right)}=\left(1000\;{\text{A}}_{470}-1.90\;{\text{C}}_{\text{a}}-63.14\;{\text{C}}_{\text{b}}\right)/214$$

### 2.3 Statistical analysis

All biochemical estimations were performed in triplicate as technical replicates (triplicate measurements represent technical rather than biological replicates), and mean values were used for subsequent analyses. Individual replicate values were not retained due to minimal technical variation. Descriptive statistics, including means, standard deviations, and frequency distributions, were calculated to summarize the data. The standard deviation (SD) was calculated using the mean values derived from technical triplicates, which represent the variability among biological samples for each trait. The correlation matrix was generated to explore the relationships between key bioactive compounds using the “Performance Analytics” R package (Version 4.2.2; Peterson et al., 2018). To perform statistical computations and data manipulation, the “Stats”, “plyr”, and “ggplot2” R packages were employed, facilitating basic statistical analyses and visualization (Kassambara, 2013; Wickham and Wickham, 2020). Pearson correlation coefficients were computed to assess linear relationships between variables, with significance levels determined through appropriate statistical tests. Strong correlations (r ≥ 0.79) were observed among vitamin C, phenols, and antioxidants, indicating interconnected metabolic pathways influencing fruit quality. Graphical representations, including bar plots, scatter plots, and heatmaps, were created using “ggplot2**”** to illustrate trends, distributions, and correlation patterns among the measured bioactive compounds. We conducted Principal Component Analysis (PCA) and hierarchical cluster analysis using R software to explore the variation and relationships among the genotypes.

## 3 Results

### 3.1 Phytochemical Analysis of capsicum genotypes across growth stages

#### 3.1.1 Comparative insights among different genotypes, colour and stage of maturity in capsicum fruits for vitamin C

**Figure 2:**
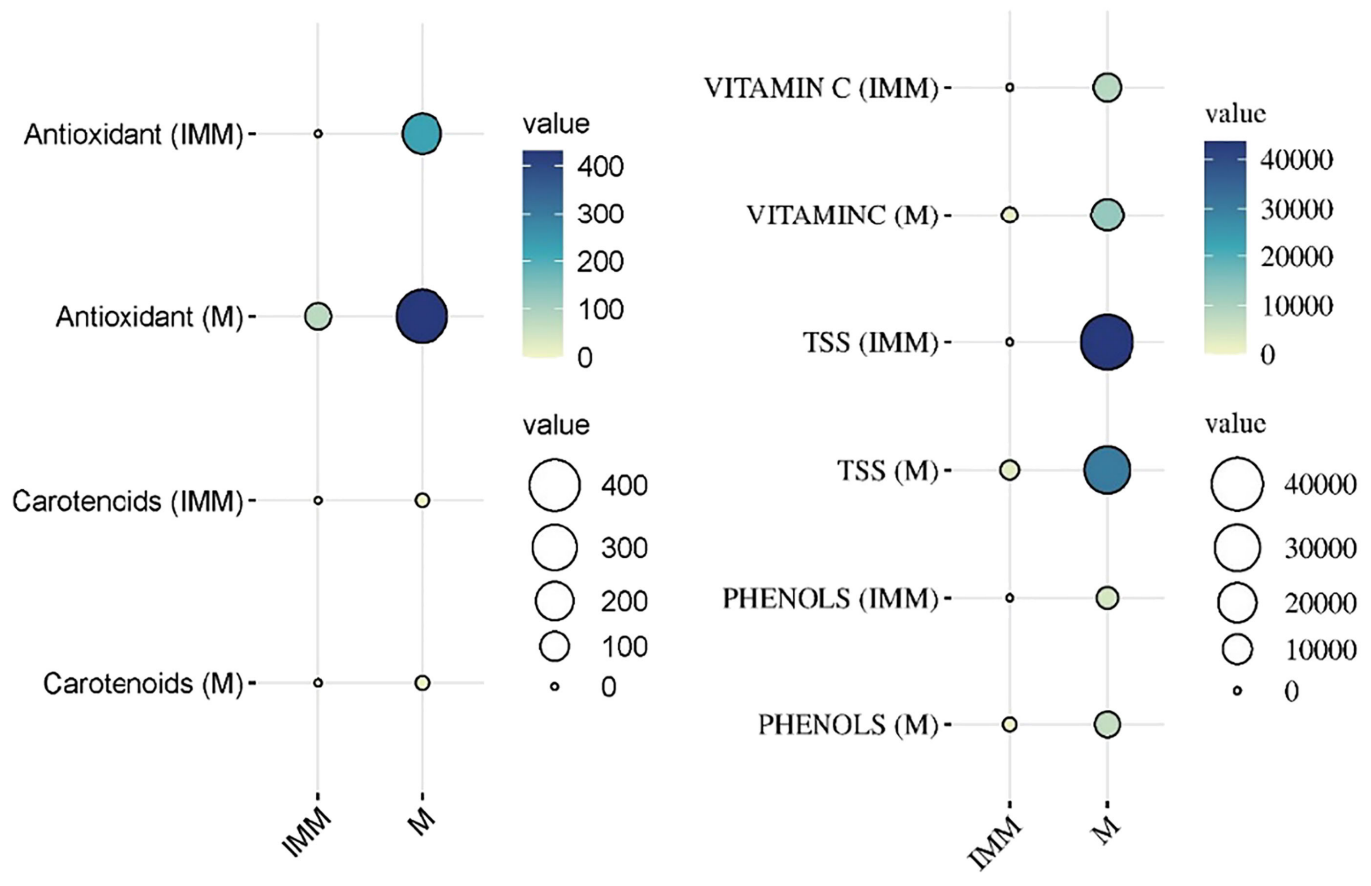
Overall evaluation of bioactive compounds across the growth stages and genotypes in capsicum.

**Figure 3:**
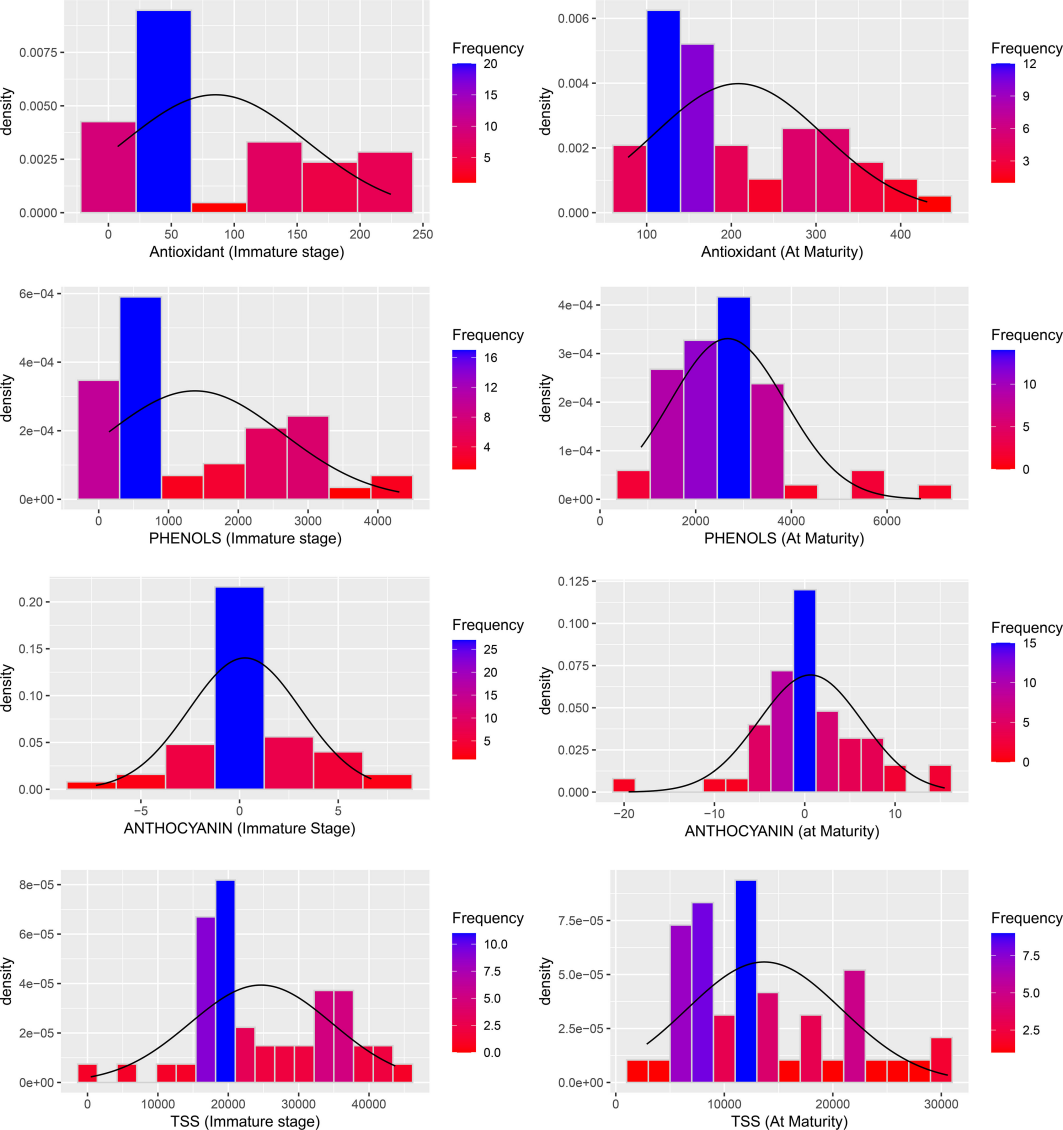
Histogram depicting phytochemical variability across the genotypes and within the stage (immature and mature) of capsicum fruits. * : Significant at 5% level (p < 0.05); ** : Significant at 1% level (p < 0.01);*** : Significant at 0.1% level (p < 0.001).

The study highlights the dynamic changes in vitamin C content in Capsicum fruits as they progress through different stages of maturation, with significant variations observed among different fruit colours and genotypes (**Figures 2**, **3)**. The data demonstrated that vitamin C levels consistently increased from the immature to the mature stage, emphasizing the potential of Capsicum fruits as an essential source of this vital antioxidant. Yellow and orange fruits, in particular, exhibited the highest vitamin C content during the immature stage, ranging from 343.00 to 8,511.72 µg/g. As these fruits matured, their vitamin C concentration rose dramatically, reaching between 1,487.72 and 12,565.95 µg/g. Among the studied genotypes, white-coloured fruits emerged as the top performers in vitamin C content at maturity, recording an impressive 12,565.95 µg/g. This remarkable concentration positions white fruits as a key candidate for future research and commercial cultivation aimed at maximizing vitamin C content in peppers. Similarly, certain red genotypes demonstrated outstanding performance in terms of vitamin C levels at maturity. For example, F4- 9(31A-5) and NBR-22 exhibited vitamin C concentrations of 11,523.07 µg/g and comparable levels to the leading white-coloured genotypes, respectively. The red genotype F5-33A-2 and the orange-coloured genotype AVR-152 demonstrated exceptional vitamin C content, emphasizing their potential as nutrient-rich Capsicum varieties. F5-33A-2 recorded a vitamin C concentration of 8,511.71 µg/g, making it a promising candidate among red genotypes for breeding programs focused on enhancing nutritional value. Similarly, the orange genotype AVR-152 performed on par with the check variety KTPL-19 across both the immature and mature stages of fruit development. Furthermore, AVR-152 outperformed several well-known commercial hybrids, including Laxmi, Indra, and Pusa Capsicum, in vitamin C content.

#### 3.1.2 Comparative insights among different genotypes, colour and stage of maturity in capsicum fruits for antioxidant

**Figure 4:**
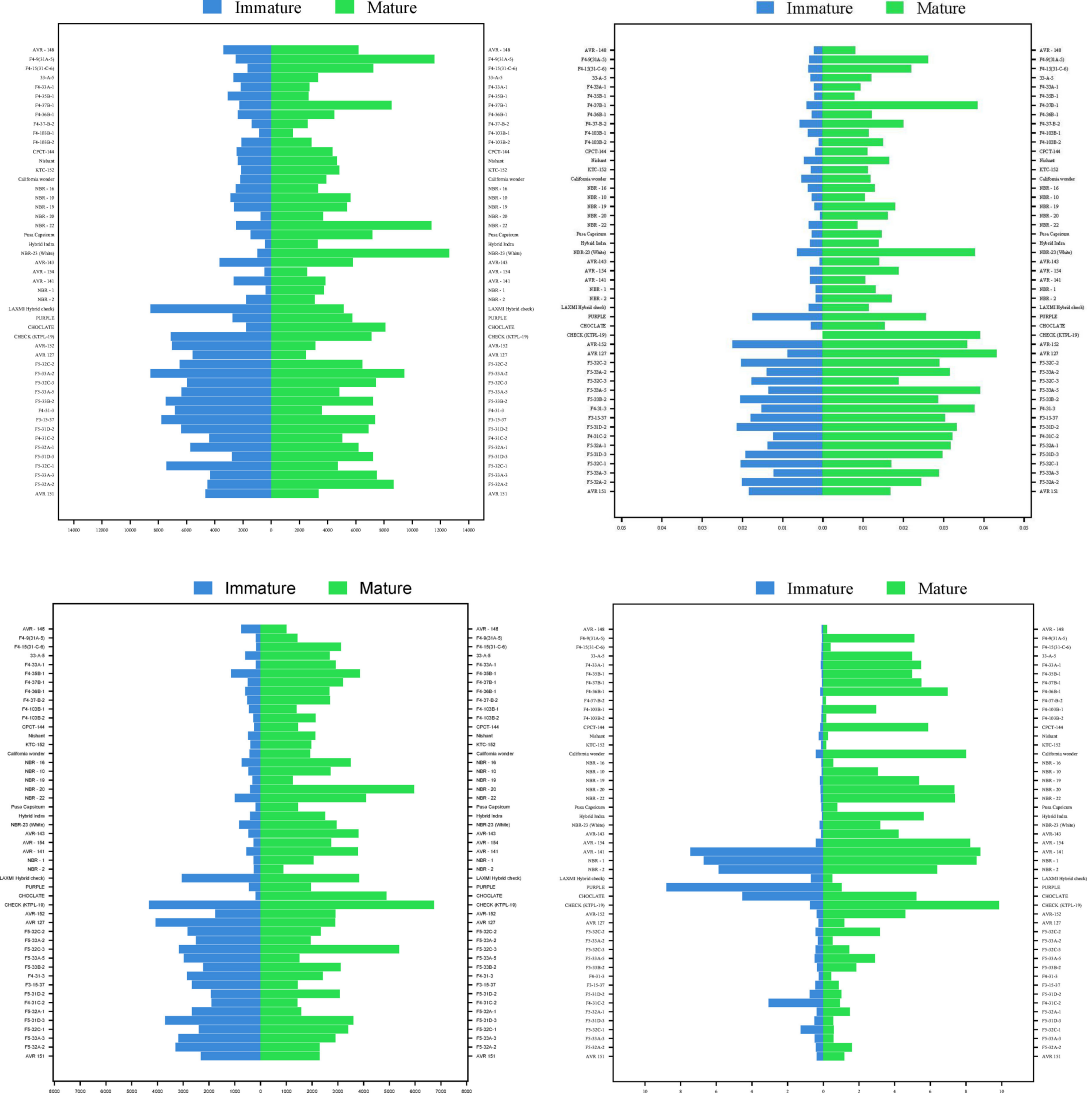
Phytochemical variability for vitamin C, antioxidant, phenols, carotenoid and xanthophyll in genotypes at immature and mature stages of sweet pepper fruit. * : Significant at 5% level (p < 0.05); ** : Significant at 1% level (p < 0.01);*** : Significant at 0.1% level (p < 0.001).

The antioxidant levels in Capsicum fruits showed a remarkable increase during the ripening process, with values ranging from 7.32 µg/g to 224.53 µg/gat the immature stage and further increasing from 78.09 µg/g to a maximum of 431.34 µg/g at the mature stage. As the data showed (**Figure 4**) among the 16 yellow genotypes examined out of 49, the majority exhibited higher antioxidant content than red genotypes at the immature stage, suggesting that these genotypes might be optimal for consumption when harvested early (**Figure 3**). However, within the red Capsicum genotypes, while antioxidant levels were generally consistent, some genotypes, such as F5-33B-2, stood out with a significantly high antioxidant content of 376.77 µg/g at the mature stage, making it a notable candidate for further research. At the mature stage, the orange-coloured genotype AVR-152 demonstrated the highest antioxidant level, reaching an impressive 431.34 µg/g, followed by the chocolate-coloured genotype with 390.31 µg/g, and F4-37B-1, which recorded 384.22 µg/g. These values were notably higher than those of popular commercial hybrids of north Indian conditions cultivation such as Laxmi, Indra, and California Wonder, indicating the superior antioxidant properties of these genotypes. Among the yellow genotypes, F5-32A-2, F5-32C-1, and F5-32C-3 were particularly noteworthy, as they exhibited significant antioxidant content at maturity, suggesting their potential as nutrient-rich options for breeding programs and dietary applications. Some genotypes exhibited a consistent level of antioxidants across both immature and mature stages, which is a unique and desirable trait.

#### 3.1.3 Comparative insights among different genotypes, colour and stage of maturity in capsicum fruits for total phenols

**Figure 5:**
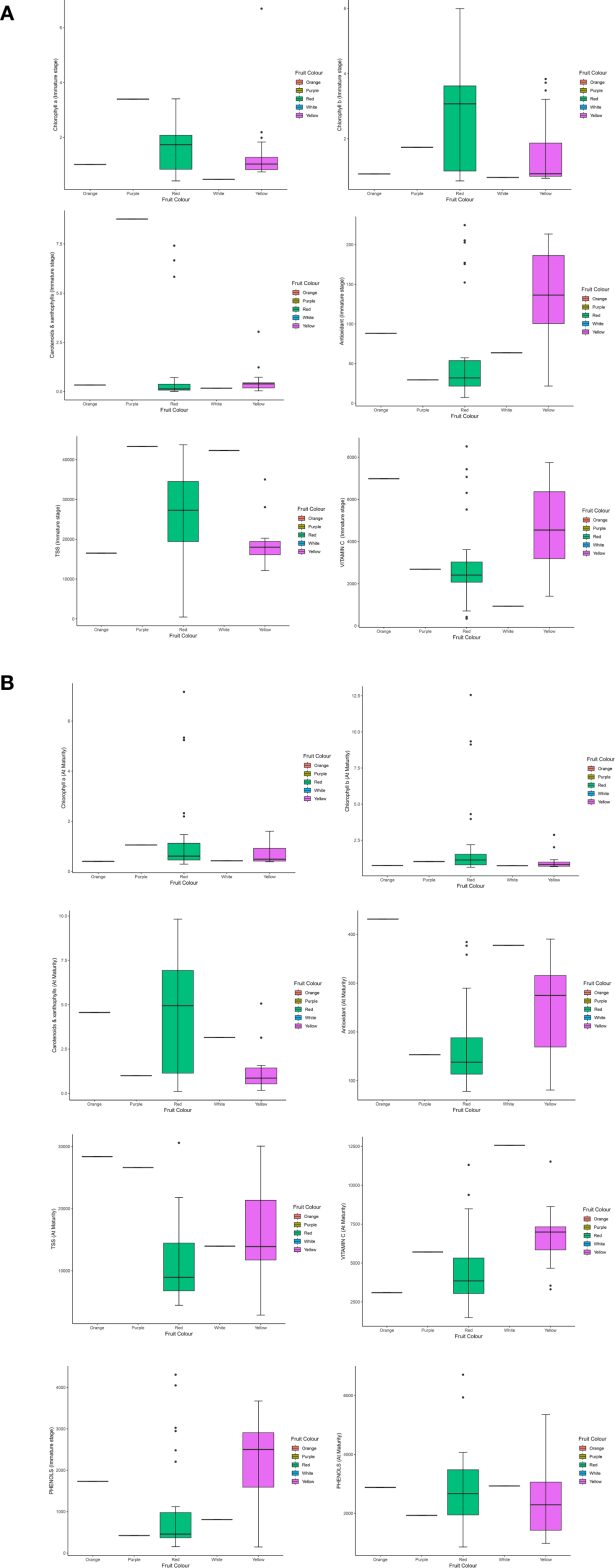
Bar graph of seven phytochemical parameters viz., vitamin C, antioxidant, Carotenoids and xanthophylls, Total Soluble Sugar, Chlorophyll and Chlorophyll b based on the colour of fruits at immature **(a)** and mature **(b)** stage.

The total phenols were observed to rise significantly, with immature Capsicum fruits showing levels ranging from 146.0 to 4308.13 µg/g, while mature fruits exhibited an even broader range, reaching 858.30 to 6707.50 µg/g. This substantial variation across different ripening stages underscores the influence of genotype and ripening phase on phenolic content. Certain genotypes displayed exceptionally high phenolic content at maturity (AVR-27, NBR-20, F5-32C-3). Among the yellow Capsicum genotypes, phenolic content was observed to be higher at the immature stage (when the fruit is green in colour), but this content substantially decreased as the fruit matured. In contrast, red genotypes exhibited lower phenolic content during the immature stage (characterized by green, purple, or white fruit colours). However, some exceptional red genotypes were identified, which recorded significantly higher total phenol levels during the immature stage of growth, particularly at the marketable size stage (**Figure 3**). These include the check variety Paprika KTPL-19, with 4308.12 µg/g, and AVR-127, which displayed levels ranging from 4048.91 µg/g to 2870.31 µg/g. Some red genotypes at maturity, consistently exhibited higher total phenolic content, followed by orange-coloured genotypes with 2879.52 µg/g, and chocolate-coloured genotypes with 4857.88 µg/g. These results demonstrate the potential of these genotypes as rich sources of phenolic compounds, contributing to their antioxidant capacity and potential health benefits. A few genotypes demonstrated high total phenol content across both stages of ripening, indicating their stability in phenolic accumulation. While yellow genotypes are more phenol-rich at the immature stage, red and chocolate-coloured genotypes dominate at maturity (**Figure 5**).

#### 3.1.4 Comparative insights among different genotypes, colour and stage of maturity in capsicum fruits for Carotenoids and Xanthophylls

The study revealed significant variability in carotenoid and xanthophyll content among different Capsicum genotypes, with values ranging from 0.01 µg/g to 7.16 µg/g during the immature stage (**Figure 1**). As the fruit matured, carotenoid content increased considerably, ranging from 0.29 µg/g to 8.77 µg/g. These results emphasize the role of maturity in enhancing the carotenoid and xanthophyll profiles of Capsicum fruits. At the immature stage, purple-coloured Capsicum displayed the highest carotenoid and xanthophyll content, with levels peaking at 8.765 µg/g, outperforming green and white-coloured fruits. Among red genotypes (AVR-141) which exhibits a green colour in its immature stage, stood out with a significant carotenoid content of 7.411 µg/g, making it a notable performer in this category. As the fruits matured, red genotypes emerged as the top contributors to carotenoid and xanthophyll content. For instance, F4-36B-1 exhibited levels ranging from 0.137 µg/g to 6.933 µg/g, while CPCT-144 and California Wonder recorded values of 0.131-5.84 µg/g and 0.375-7.95 µg/g, respectively. These results compare favourably with the check variety Paprika KTPL-19, which displayed the highest carotenoid content at maturity, ranging from 0.714 µg/g to 9.821 µg/g, and the hybrid Laxmi, which showed levels of 0.651 µg/g to 0.472 µg/g. Several genotypes exhibited consistent carotenoid and xanthophyll content across both stages of maturity, indicating their stability in these compounds. Additionally, Chocolate-coloured Capsicum maintained steady levels of 4.499-5.187 µg/g, further highlighting the potential of these genotypes for consistent carotenoid and xanthophyll production. In conclusion, the study underscores the variation in carotenoid and xanthophyll content across different genotypes and ripening stages of Capsicum. While purple-coloured fruits excel at the immature stage, red genotypes dominate at maturity are promising candidates for breeding programs targeting enhanced nutritional profiles in Capsicum.

#### 3.1.5 Comparative insights among different genotypes, colour and stage of maturity in capsicum fruits for Total soluble sugar

**Figure 6:**
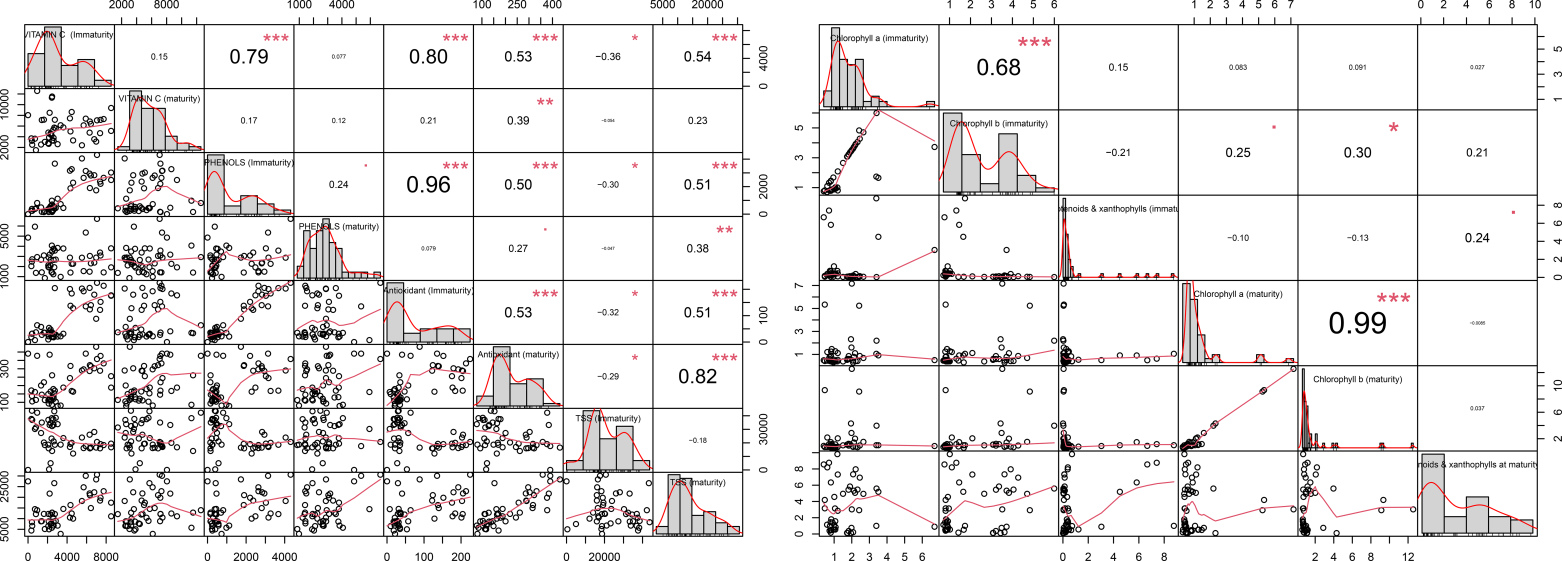
Pearson correlation coefficients of capsicum genotypes for phytochemical compounds studied at different stages of maturity. * : Significant at 5% level (p < 0.05); ** : Significant at 1% level (p < 0.01);*** : Significant at 0.1% level (p < 0.001).

The total soluble sugar (TSS) content in Capsicum fruits demonstrates significant variation between the immature and mature stages of fruit development, with a clear trend of decreasing TSS levels as the fruit ripens. TSS content ranges from a high of 43,730.5 µg/g in the immature stage to 27,748.23 µg/g at full maturity (**Table 1**). This indicates that Capsicum fruits are generally sweeter during the immature stage compared to their mature counterparts. In terms of fruit colour, red and purple genotypes exhibited the highest TSS content during the immature stage, followed by white and orange genotypes (**Figure 5**). Outstanding performers at the immature stage included Hybrid Indra, which recorded the highest TSS content at 43,730.57 µg/g, and purple genotype (43,326.03 µg/g). Other notable genotypes with elevated TSS levels in the immature stage were NBR-23 (White) (42,277.97 µg/g), AVR-154 (36,902.65 µg/g), NBR-1 (37,651.29 µg/g), AVR-141 (33,282.4 µg/g), and KTC-152 (36,979.09 µg/g). At maturity, orange and yellow genotypes displayed relatively higher TSS content, showcasing a shift in sugar accumulation patterns with ripening (**Figure 6**). Certain genotypes maintained their elevated TSS levels even as the fruit matured, defying the general trend of decline. These included Chocolate genotype (27,483.07 µg/g), AVR-152 (28,385.11 µg/g), F5-32C-3 (30,073.44 µg/g), and F5-32A-1 (24,689.24 µg/g). Their consistently high TSS levels at maturity make them suitable candidates for processing and culinary applications where sweetness is a desired trait. In contrast, California Wonder, a widely cultivated commercial variety, exhibited the lowest TSS values at both the immature and mature stages, indicating a relatively less sweet profile compared to other genotypes. The observed variation in TSS content across developmental stages and fruit colours underscores the importance of genotype selection in breeding programs (**Figure 5**). Genotypes such as Hybrid Indra, Purple, AVR-152, and F5-32C-3 offer significant potential for developing Capsicum varieties tailored to specific market preferences, whether for high sweetness at the immature stage or consistent sugar content at full maturity.

**Table 1:** Descriptive statistics of bioactive compounds across growth stages in sweet peppers.

Estimates	Chl a (I)	Chl b (I)	C + X (I)	C + X (M)	Chl b (M)	C + X (M)	vitamin C (I)	Phenols (I)	Antioxidant (I)	TSS (I)	Vitamin C (M)	Phenols (M)	Antioxidant (M)	TSS (M)
Min.	0.41	0.71	0.01	0.29	0.62	0.11	343.00	0.00	7.32	0.00	1487.72	858.30	78.09	2850.79
Max.	6.70	5.99	7.16	8.77	12.55	9.82	8511.72	4308.13	224.53	43730.57	12565.95	6707.50	431.34	30599.02
Range	6.29	5.29	8.76	6.87	11.93	9.72	8511.72	4308.13	224.53	43730.57	11078.24	5849.20	353.24	27748.23
Sum	77.27	105.87	47.20	51.65	85.28	160.43	175166.09	66129.61	4086.29	1183148.00	268075.25	132979.80	10393.23	683739.32
Median	1.19	1.46	0.24	0.60	0.96	2.85	2621.72	571.08	37.00	20679.50	4990.62	2654.88	170.74	12198.54
Mean	1.58	2.16	0.96	1.05	1.74	3.27	3574.82	1349.58	83.39	24145.87	5470.92	2713.87	212.11	13953.86
SE(Mean)	0.16	0.21	0.29	0.19	0.34	0.42	342.86	180.55	10.37	1517.71	362.13	176.22	14.63	1049.00
CI.mean.0.95	0.31	0.42	0.59	0.39	0.68	0.83	689.36	363.03	20.85	3051.56	728.12	354.30	29.42	2109.15
Var.	1.19	2.11	4.16	1.82	5.66	8.42	5760016.24	1597361.67	5268.32	112868900.00	6425843.91	1521536.43	10488.18	53919257.27
Std. Dev	1.09	1.45	2.04	1.35	2.38	2.90	2400.00	1263.87	72.58	10623.98	2534.93	1233.51	102.41	7342.97
Coef. Var	0.69	0.67	2.12	1.28	1.37	0.89	0.67	0.94	0.87	0.44	0.46	0.46	0.48	0.53

Immature stage; M- Mature stage; chl a-Chlorophyll a; chlo b- Chlorophyll b; C + X; Carotenoids and Xanthophyll; TSS- Total Soluble Sugar; Min. – Minimum; Max. – Maximum; Range = (Maximum − Minimum); Mean – Arithmetic Mean (Average); SE(Mean) – Standard Error of Mean; CI.mean.0.95 – 95% Confidence Interval of the Mean; Var. – Variance; Std. Dev – Standard Deviation; Coef. Var – Coefficient of Variation (CV%)

#### 3.1.6 Comparative insights among different genotypes, colour and stage of maturity in capsicum fruits for Anthocyanin

The presence of anthocyanin in capsicum fruits varies significantly across genotypes and developmental stages, with the highest levels recorded in purple genotypes during the immature stage (**Supplementary Table S2**). Specifically, the genotype purple genotype (**Figure 2**) exhibited the highest anthocyanin content at immaturity, reaching 15.49 µg/g, but experienced a dramatic decrease during the ripening process, ultimately declining to -0.40077 µg/g at maturity. This decline reflects a common trend where anthocyanin levels diminish as fruits progress toward full ripeness. This negative value does not represent an actual decrease below zero anthocyanin; instead, it results from data standardization (e.g., mean-centring or Z-score transformation), where values lower than the population mean appear as negative numbers. Thus, negative values should not be replaced with zero, as they retain essential statistical information and reflect relative differences, not absolute concentrations.

Among the red genotypes, anthocyanin was detected at both immature and mature stages. The check variety KTPL-19 recorded the second-highest anthocyanin content at the immature stage (14.36 µg/g), making it a noteworthy genotype for anthocyanin pigmentation. Other significant red genotypes (**Supplementary Table S1**.) include AVR-148: 8.1 µg/g (immature stage), F4-103B-1: 9.21 µg/g (immature) and 5.8 µg/g (mature), F4-103B-2: 7.61 µg/g (immature) and 1.7 µg/g (mature), California Wonder: 7.34 µg/g (immature) and 4.84 µg/g (mature), AVR-154: 5.67 µg/g (immature) and 3.00 µg/g (mature), F4-33A-1 and NBR-22: 4.00 µg/g at immaturity, with levels dropping to -0.1 µg/g at maturity. The data reveal that anthocyanin levels generally decline during ripening across all genotypes, but red genotypes retain a relatively higher amount of pigmentation at maturity compared to others. Interestingly, yellow genotypes did not exhibit any anthocyanin pigmentation at either stage of fruit maturity. This absence of anthocyanin highlights a clear distinction in pigmentation characteristics based on genotype and colour.

#### 3.1.7 Comparative insights among different genotypes, colour and stage of maturity in capsicum fruits for chlorophyll a and b

Chlorophyll content in Capsicum fruits varies significantly across developmental stages and genotypes, with distinct trends for chlorophyll a and b (**Figure 3**). At the immature stage, among coloured Capsicum, purple genotypes (3.39 µg/g), exhibited the most significant chlorophyll a content, followed by red, yellow, and orange genotypes emphasizing the role of chlorophyll in photosynthetic activity during early fruit development. However, an exception was noted with the yellow genotype F4-31C-2, which recorded the highest chlorophyll a content (6.69 µg/g) at immaturity. In the mature stage, red genotypes dominated chlorophyll a level, followed by purple genotypes, showing a shift in chlorophyll distribution as the fruit ripens. The genotype NBR-10 stood out, recording the highest chlorophyll a content (7.16 µg/g) at maturity, followed by AVR-143 (5.33 µg/g) and F4-103B-1 (5.23 µg/g. At maturity, red genotypes again led in chlorophyll b levels, followed by purple genotypes, mirroring the trend observed for chlorophyll a. Notably, NBR-10 was again the top performer, with the highest chlorophyll b content (12.55 µg/g), followed by AVR-143 (9.34 µg/g) and F4-103B-1 (9.12 µg/g). The robust chlorophyll content in NBR-10 at both stages solidifies its position as a key genotype for further research, particularly for its potential role in enhancing photosynthetic activity and nutrient assimilation in Capsicum.

### 3.2 Correlation study of fruit colour and biochemical parameters across growth stages

#### 3.2.1 Yellow capsicum

At immature stage, vitamin C content positively correlates with antioxidant levels (0.601), indicating that higher vitamin C is associated with higher antioxidant levels at this stage whereas vitamin C is negatively correlated with maturity (-0.026), suggesting a slight decline as the fruit matures (**Table 2**). Antioxidants increase with maturity (0.672), which implies that antioxidants accumulate as the yellow peppers ripen, even though vitamin C decreases. During green colour of fruits or at immaturity negatively correlates with antioxidants (-0.105), which may imply that early-stage sweetness does not necessarily correspond with antioxidant presence. Negatively correlated with TSS (-0.254) and vitamin C (-0.263), suggesting that as phenols increase, sweetness (TSS) and vitamin C decrease. This trade-off indicates that phenols might dominate at the expense of other nutrients during early fruit development (immaturity). TSS established a positive correlation between immature and mature stages (0.440), showing a consistent trend where sweetness levels build up as the fruit ripens. TSS is high and positively correlated with antioxidant levels (0.886), meaning that sweetness (as indicated by TSS) aligns with higher antioxidant levels at maturity. Antioxidants were highly correlated with TSS at the mature stage (0.886). This highlights that sweetness and antioxidant levels peak together during ripening, making mature yellow capsicum an excellent choice for combined flavour and health benefits.

**Figure 7:**
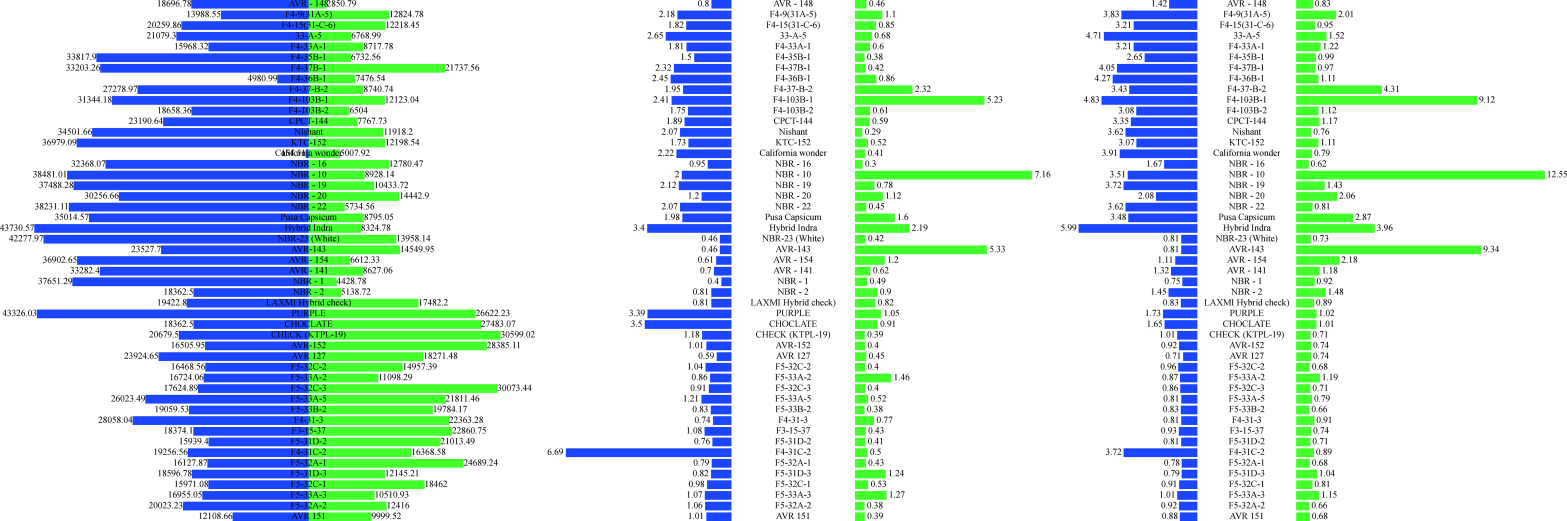
The phytochemical variability for total soluble sugar, Chlorophyll a and b in genotypes at immature and mature stages of sweet pepper fruit. * : Significant at 5% level (p < 0.05); ** : Significant at 1% level (p < 0.01);*** : Significant at 0.1% level (p < 0.001).

**Table 2:** Correlation analysis of phytochemical compounds at different maturity stages in yellow and red fruit colour of capsicum, for vitamin C, phenols, antioxidants and TSS at immature and mature growth stages.

Parameters	Vitamin C (Immature stage)		Phenols (Immature stage)		Antioxidant (Immature stage)		TSS (Immature stage)		Vitamin C (At maturity)		Phenols (At maturity)		Antioxidant (At maturity)		TSS (At maturity)	
Colour	Yellow	Red	Yellow	Red	Yellow	Red	Yellow	Red	Yellow	Red	Yellow	Red	Yellow	Red	Yellow	Red
Immature stage																
Vitamin C	1	1														
Phenols	0.602*	0.843***	1	1												
Antioxidant	0.601*	0.861***	0.921***	0.959***	1	1										
TSS	-0.291	-0.397*	-0.254	-0.23	-0.203	-0.283	1	1								
Maturity																
Vitamin C	-0.403	0.297	-0.263	0.221	-0.219	0.255	-0.094	0.172	1	1						
Phenols	0.189	0.209	0.448	0.364*	0.229	0.202	-0.174	0.052	-0.036	0.218	1	1				
Antioxidant	0.672**	0.489**	0.336	0.601***	0.394	0.655***	-0.22	-0.003	-0.026	0.502**	0.378	0.271	1	1		
TSS	0.685**	0.605***	0.44	0.728***	0.484	0.729***	-0.105	-0.023	-0.104	0.309	0.451	0.475**	0.886***	0.815***	1	1

* : Significant at 5% level (p < 0.05); ** : Significant at 1% level (p < 0.01); *** : Significant at 0.1% level (p < 0.001).

#### 3.2.2 Red capsicum

In red capsicum, the vitamin C and antioxidant showed a strong positive correlation (0.861), indicating that as vitamin C content increases, antioxidant levels also increase significantly in immature red peppers. Whereas at a mature stage, a moderate positive correlation (0.489) was established between vitamin C and antioxidants (**Table 2**), suggesting that this relationship is somewhat maintained as the capsicum mature, though less intensely than at the immature stage. At the immature Stage, TSS and antioxidants positively correlated (0.729), indicating that higher sweetness in immature red peppers aligns with antioxidant presence. While at the mature stage, even stronger positive correlation (0.815), showing that in mature red peppers, both sweetness and antioxidants are highly associated. The antioxidants are robustly linked (0.601), to biochemical changes at the immature stage, making immature red peppers an excellent source of antioxidants (Choi et al., 2023). Total phenolic compounds are more dominant in the immature stage and decline as the fruit ripens (0.364), on the other hand, total phenols negatively correlated with TSS during the immature stage. This suggests that higher phenols might limit the accumulation of sugars in young red peppers. The Total soluble sugar at maturity of capsicum fruit was strongly correlated with TSS (0.728), indicating significant sugar build-up, which aligns with the fruit’s increased sweetness and overall nutritional value as it ripens.

#### 3.2.3 Correlation study of genotypes at different Maturity stages with different phytochemical content.

At the immature growth stage, most genotypes exhibit a high correlation between vitamin C and phenols (r = 0.79) (**Figure 7**), as well as between antioxidants and vitamin C (r = 0.80) (**Tables 3**, **4**). Antioxidants also show a strong positive correlation with phenols (r = 0.96). For chlorophyll pigments, chlorophyll a and chlorophyll b are moderately correlated at the immature stage (r = 0.68). However, at the mature stage, this association becomes much stronger (r = 0.99), indicating a tighter relationship between the two pigments as fruits advance toward full ripening. Carotenoid and xanthophyll levels also increase substantially with maturity across nearly all genotypes. Nevertheless, variation exists both across maturity stages and among genotypes, and not all genotypes follow the same phytochemical accumulation pattern.

**Table 3:** Correlation analysis of phytochemical compounds at different maturity stages in yellow and red fruit colour of capsicum, for Chlorophyll a, Chlorophyll b and Carotenoids & xanthophylls at immature and mature growth stages.

Compounds	Chlorophyll a (Immature stage)		Chlorophyll b (Immature stage)		Carotenoids & xanthophylls (Immature stage)		Chlorophyll a (At maturity)		Chlorophyll b (At maturity)		Carotenoids & xanthophylls (At maturity)	
Colour/Stage	Yellow	Red	Yellow	Red	Yellow	Red	Yellow	Red	Yellow	Red	Yellow	Red
Immature stage												
Chlorophyll a	1	1										
Chlorophyll b	0.714**	0.974***	1	1								
Carotenoids & xanthophylls	0.81***	-0.413*	0.23	-0.346	1	1						
Maturity stage												
Chlorophyll a	0.048	0.138	0.453	0.191	-0.252	-0.147	1	1				
Chlorophyll b	0.184	0.163	0.671**	0.224	-0.221	-0.146	0.825***	0.994***	1	1		
Carotenoids & xanthophylls	0.072	0.031	0.297	0.051	-0.154	0.417*	-0.001	-0.126	0.239	-0.105	1	1

* : Significant at 5% level (p < 0.05); ** : Significant at 1% level (p < 0.01); *** : Significant at 0.1% level (p < 0.001).

**Table 4:** Correlation analysis of phytochemical compounds at different maturity stages of sweet pepper fruits across all colours.

Stage	Compounds	Immature			Mature			Immature				Mature			
		Chlorophyll a	Chlorophyll b	C+X	Chlorophyll a	Chlorophyll b	C+X	Vitamin C	Phenols	Antioxidant	TSS	Vitamin C	Phenols	Antioxidant	TSS
Immature	Chlorophyll a	1.000													
	Chlorophyll b	0.678***	1.000												
	C+X	0.155	-0.207	1.000											
Mature	Chlorophyll a	0.083	0.255	-0.104	1.000										
	Chlorophyll b	0.091	0.302*	-0.131	0.988***	1.000									
	C+X	0.027	0.212	0.244	-0.008	0.037	1.000								
Immature	VITAMIN C	-0.319*	-0.572***	-0.179	-0.215	-0.253	-0.369**	1.000							
	PHENOLS	-0.332*	-0.619***	-0.183	-0.246	-0.298*	-0.324*	0.795***	1.000						
	Antioxidant	-0.285*	-0.589***	-0.188	-0.221	-0.280	-0.383**	0.799***	0.957***	1.000					
	TSS	0.009	0.209	0.121	0.233	0.272	0.072	-0.36*	-0.297*	-0.317*	1.000				
Mature	VITAMIN C	-0.037	-0.165	-0.093	-0.118	-0.165	-0.090	0.152	0.173	0.211	-0.054	1.000			
	PHENOLS	-0.173	-0.229	-0.043	-0.039	-0.045	0.291*	0.077	0.241	0.079	-0.047	0.119	1.000		
	Antioxidant	-0.063	-0.438**	-0.037	-0.264	-0.283*	-0.175	0.532***	0.505***	0.534***	-0.288*	0.387**	0.275	1.000	
	TSS	-0.012	-0.432**	0.081	-0.160	-0.187	-0.185	0.544***	0.512***	0.51***	-0.180	0.229	0.382**	0.823***	1.000

C + X; Carotenoids and Xanthophyll; TSS- Total Soluble Sugar. * : Significant at 5% level (p < 0.05); ** : Significant at 1% level (p < 0.01); *** : Significant at 0.1% level (p < 0.001).

### 3.3 Principal component analysis and hierarchical clustering

A principal component analysis (PCA) was conducted (**Supplementary Figure S1**) to explore the relationships among genotypes based on biochemical traits, and it showed considerable variability in traits contributing to the Dim1, particularly antioxidant, total phenols, vitamin C, and TSS at the IMM stage, which had high positive loadings along this component. In turn, this component is represented the overall biochemical richness at the immature stage. Carotenoids & Xanthophyll at both maturity stages showed negative loadings on Dim1 and Dim2, suggesting an inverse relationship with antioxidant and phenolic traits.

Hierarchical cluster analysis was performed to examine the genetic relationships among the tested genotypes. The resulting dendrogram (**Supplementary Figure S2**) grouped the 49 genotypes into four major clusters, indicating clear differentiation among them. The clustering pattern suggests substantial genetic variation among the tested genotypes, which could be further exploited for selection in breeding programs targeting the traits under study.

## 4 Discussion

### 4.1 Vitamin C and antioxidant

The maturity stage, genotype, environment, processing and storage conditions are some of the variables that can affect the phytochemicals present in fruits and vegetables (Lightbourn et al., 2008). Peppers are a rich source of ascorbic acid (vitamin C), containing concentrations that exceed three times the recommended dietary allowance (Howard et al., 2000). This study underscores the dynamic changes in vitamin C levels throughout the maturation stages of peppers, highlighting the importance of genotype selection for breeding programs targeting nutrient-rich cultivars.

The concentration of vitamin C generally increases as peppers mature, consistent with previous findings that ripening enhances ascorbic acid content (Chávez-Mendoza et al., 2015). Among the different colours of peppers, yellow peppers exhibit the highest vitamin C levels, followed by orange peppers. Some genotypes display a more complex pattern of ascorbic acid accumulation like show a rise in vitamin C content at the onset of ripening, followed by a gradual decline as ripening progresses (AVR-127, AVR-152, and F4-33A-5). This pattern is attributed to the antioxidant role of vitamin C, which becomes more active during the increased respiration rates associated with the climacteric phase of fruit development (Mateos et al., 2013; Zhigila et al., 2014).

Vitamin C levels in peppers vary significantly across species and genotypes. For instance, *Capsicum chinense* genotypes exhibit a wide range of vitamin C content (54.1–129.8 mg/100 g), whereas *Capsicum annuum* ranges from 101 to 114.85 mg/100 g (Teodoro et al., 2013; Perucka and Materska, 2007). Hybrid varieties like Orobelle, Bomby, and Paladin show even higher concentrations, with Orobelle recording 111.97 mg/100 g (Kumar et al., 2015). Notably, Habanero peppers (*Capsicum chinense*) are particularly rich in ascorbic acid, with concentrations ranging from 187.24 to 281.73 mg/100 g (Majidi and Hazim, 2016). The findings suggest that the optimal stage for consuming peppers to maximize vitamin C intake varies depending on the genotype. For instance, immature stages of certain genotypes were rich in ascorbic acid, while fully ripe yellow peppers provide the highest levels of vitamin C among colour variants. Understanding these variations can guide consumer choices and inform agricultural practices to maximize the nutritional value of pepper crops. In conclusion, vitamin C content in peppers is influenced by a complex interplay of genetic, environmental, and developmental factors. Identifying genotypes with consistently high ascorbic acid levels, such as CPCT-31A-5 and NBR-22, offers opportunities for breeding nutrient-dense pepper varieties.

The antioxidant components and activity of sweet peppers of the green, yellow, orange, and red varieties (*Capsicum annuum* L.) have been the subject of numerous investigations. As the bell pepper ages, its colour changes from green to a variety of shades, including orange, red, and yellow (Choi et al., 2023). The evaluation of antioxidant content across these various colour variants of 49 genotypes at two stages of maturity was the main focus of this study. With considerable genotype and colour-related differences, these investigations showed that the antioxidant content is relatively high in the genotypes that are red at the mature stage (Hervert-Hernandez et al., 2010; Park et al., 2012). According to the research, choosing particular genotypes may improve the nutritional content of capsicum fruits, especially with regard to vitamin C and antioxidant content (Nadeem et al., 2011). However, research revealed that antioxidants in red peppers have high relationships at both the immature and mature stages, whereas, in yellow peppers, antioxidants are associated with total soluble sugar at the mature stage. This makes red peppers a more consistent antioxidant source across maturity stages.

### 4.2 Total phenols

Phenolic content varied markedly across colours and stages, with immature yellow fruits exhibiting higher phenolics that declined with ripening. Mature red peppers retained the overall highest phenolic content, consistent with their strong free radical scavenging capacity. Strong correlations between phenols and antioxidants (r = 0.96) reaffirm phenolics as major contributors to antioxidant activity (Steelheart et al., 2020). These results also support the role of phenylpropanoid-derived phenols in pigmentation and stress responses during ripening.

### 4.3 Carotenoids and xanthophyll

Carotenoids and xanthophylls are major determinants of pepper colour and nutritional value, providing strong antioxidant activity that helps reduce oxidative stress (Ha et al., 2007). These pigments are primarily stored in chromoplast fibrils, with smaller amounts freely dispersed. Their concentration varies across genotypes, with red peppers showing the highest carotenoid levels at full maturity due to the accumulation of red ketocarotenoids such as capsanthin and capsorubin (Biacs et al., 1993; Biacs et al., 1989). These pigments are uniquely synthesized in *Capsicum* through the enzyme capsanthin–capsorubin synthase (CCS), which converts 5,6-epoxycarotenoids into red pigments via a pinacol rearrangement (Deli et al., 2001). During ripening, chlorophyll degradation and chromoplast development drive the transition from green to yellow, orange, and red. β-carotene dominates in orange fruits, while yellow and some orange cultivars accumulate violaxanthin and β-cryptoxanthin due to CCS gene absence or inactivation (Lefebvre et al., 1998; Lang et al., 2004; Huh et al., 2001; Guzman et al., 2010). Expression of key carotenoid biosynthesis genes such as *Psy, Pds, CrtZ-2*, and *Ccs* increases during ripening, corresponding to pigment accumulation; mutations in these genes significantly alter colour phenotypes (Ha et al., 2007; Romero-da Cruz et al., 2017). Environmental conditions—light, temperature, and soil quality—further influence carotenoid synthesis (Collera-Zúñiga et al., 2005). Major carotenoids including β-carotene, capsanthin, lycopene, zeaxanthin, and lutein are recognized for their health-promoting properties and potential in preventing chronic degenerative diseases.

### 4.4 Total soluble sugar

Total soluble sugar (TSS) levels in *Capsicum* vary notably between developmental stages, with many genotypes showing higher TSS in the immature stage before declining at maturity. This contrasts with reports such as Pingping et al. (2022), who observed increasing sugars during ripening, suggesting strong genotype-dependent differences. Some genotypes—like Chocolate, AVR-152, and F5–32 lines—retain high TSS even at maturity, while others, such as California Wonder, exhibit consistently low sweetness (Irigoyen et al., 1992). TSS also interacts with antioxidant content in a colour- and stage-specific manner: mature yellow peppers show a strong TSS–antioxidant relationship, immature yellow peppers show weak alignment, and red peppers maintain a stable correlation across stages. These results reveal the importance of genotype and ripening stage in determining sweetness and nutritional quality (Bosland and Votava, 2012; Ghasemnezhad et al., 2011).

### 4.5 Anthocyanin

Anthocyanins, the final products of the flavonoid pathway, are key pigments responsible for purple and black colouration in Capsicum fruits. They accumulate mainly in the outer epidermis and include derivatives such as delphinidin, cyanidin, and malvidin, with levels varying across genotypes and developmental stages (Lightbourn et al., 2008). In the present study, the Purple genotype showed high anthocyanin content at the immature stage (8.76 µg/g), which declined sharply to 0.997 µg/g upon ripening. The reduction in anthocyanins during maturation may result from decreased biosynthesis or increased degradation. While pH-mediated structural changes occur *in vitro* (Basílio and Pina, 2016), in plants, the decline is typically due to reduced biosynthetic activity or enhanced breakdown (Borovsky et al., 2004). Similar trends are reported in other Solanaceae crops like tomato and eggplant, where anthocyanins peak in unripe fruits but diminish as they ripen (Mennella et al., 2012). Chlorophyll can mask anthocyanin pigmentation in immature green fruits. With ripening, chlorophyll degrades and underlying pigments become visible; however, anthocyanin biosynthesis often slows due to downregulation of genes like CaMYBA and other structural genes (Borovsky et al., 2004). Certain genotypes (e.g., AVR-141, NBR-1, NBR-2) maintained high anthocyanin levels even at maturity, indicating their potential as anthocyanin-rich varieties. This contrasts with most purple genotypes that show sharp declines during ripening. The inconsistent relationship between anthocyanin and carotenoid content across genotypes highlights the complex interaction of pigment pathways, influenced by regulatory genes such as ANT1 and SlAN2, which modulate the activation of downstream structural genes (Jarret et al., 2019; Niu et al., 2017).

### 4.6 Chlorophyll a and b

In the present study, the genotype NBR-10 emerged as the top performer in chlorophyll content at the mature stage. It recorded the highest chlorophyll b level (12.54 µg/g), followed by AVR-143 and F4-103B-1. A similar trend was observed for chlorophyll a, where NBR-10 again ranked highest, highlighting its potential for studies related to photosynthetic efficiency and yield improvement. At the immature stage, most genotypes exhibited higher chlorophyll b than chlorophyll a, reflecting the functional role of chlorophyll b in broadening the light-harvesting range during early developmental stages (Lichtenthaler, 1987; Porra et al., 1989). Chlorophyll content is a critical indicator of photosynthetic capacity, fruit maturity, and overall quality. Variations in chlorophyll a and b across genotypes emphasize the scope for breeding programs aimed at enhancing both nutritional attributes and visual appeal in Capsicum fruits. Given its consistently high pigment levels, NBR-10 represents a promising candidate for future research targeting improved photosynthetic efficiency and the development of high-performing pepper cultivars (Gross, 1991; Taiz & Zeiger, 2003).

In order to explain the population dynamics, PCA revealed substantial biochemical variability among the 49 genotypes. Dim1 was mainly influenced by antioxidant activity, total phenols, vitamin C, and TSS at the immature stage, reflecting overall biochemical richness, while carotenoids and xanthophylls showed negative loadings, indicating an inverse relationship with antioxidant traits. Hierarchical cluster analysis grouped the genotypes into four distinct clusters, highlighting significant genetic variation. The clustering aligns with PCA results, suggesting that divergent genotypes could be selected as parents for breeding programs aimed at improving nutritional and bioactive traits across maturity stages.

## 5 Conclusions

Overall, the findings underscore the importance of genotype selection in the development of nutrient-dense *Capsicum* varieties. By identifying and leveraging high-performing genotypes, breeders can address the dual goals of enhancing nutritional quality and meeting consumer preferences. This study’s unique, comprehensive analysis of different sweet pepper colours—red, yellow, orange, white, purple, and chocolate—across two maturity stages provides critical insights, as such an integrated approach has not been reported previously. The results reveal that bioactive compounds are significantly influenced by both the stage of maturity and the fruit colour, with notable variability among genotypes. However, certain genotypes exhibited consistent bioactive compound concentrations at both maturity stages, making them particularly valuable for breeding and consumption. The high point of this work lies in its ability to provide a holistic understanding of how genotype, fruit colour, and maturity stages interact to influence the nutritional and functional quality of *Capsicum*. This comprehensive approach not only fills a significant gap in the existing literature but also offers practical insights for targeted breeding, post-harvest management, and consumer health recommendations. For consumers, mature yellow peppers are recommended for sweetness and antioxidants, while immature red peppers are ideal for antioxidant and phenol-rich options. Notably, immature purple capsicum showed the highest anthocyanin content, while mature white capsicum exhibited the highest vitamin C levels. Future research into secondary metabolites, enzymatic pathways, and regulatory mechanisms will further support the development of *Capsicum* varieties with optimized nutritional value, aesthetic appeal, and environmental adaptability. This work sets a foundation for future studies and breeding programs aimed at enhancing the health benefits and commercial value of sweet peppers globally.

## Data Availability

The original contributions presented in the study are included in the article/**Supplementary Material**. Further inquiries can be directed to the corresponding authors.

## Supplementary material

Supplementary Table 1 Mean values of vitamin C, total phenols (µg/g), antioxidant (µg/g), total solid sugars (TSS) (µg/g), carotenoids & Xanthophyll (C + X) (µg/g) and Chlorophyll a and b of capsicum genotypes used in this study. *C+X- Carotenoids & Xanthophyll

Supplementary Table 2 Mean of anthocyanin µg/g during different growth stages of capsicum.

Supplementary Figure 1 Principal Component Analysis (PCA) biplot showing the distribution of genotypes based on seven biochemical traits at immature and mature growth stages.

Supplementary Figure 2 Hierarchical clustering dendrogram. The dendrogram provides a visual representation of how the Capsicum genotypes in the dataset are grouped into clusters based on their similarities in the biochemical parameters.
