# Supplementary material for: Phytochemical characterization and correlation analysis of nutritional value in sweet pepper (Capsicum annuum L.) genotypes at various growth stages
Source: Front Plant Sci. 2026 Feb 11;16:1719537. doi: 10.3389/fpls.2025.1719537 (PMC12932425; doi:10.3389/fpls.2025.1719537)
Supplement: Supplementary Table 1 — Mean values of vitamin C, total phenols (µg/g), antioxidant (µg/g), total solid sugars (TSS) (µg/g), carotenoids & Xanthophyll (C + X) (µg/g) and Chlorophyll a and b of capsicum genotypes used in this study. *C+X- Carotenoids & Xanthophyll [file Table1.docx]

**Supplementary Table 1.** Mean values of vitamin C, total phenols (µg/g), antioxidant (µg/g), total solid sugars (TSS) (µg/g), carotenoids & Xanthophyll (C + X) (µg/mL) and Chlorophyll a and b of capsicum genotypes used in this study.

| **S. No.** | **Genotype** | **Fruit Colour** | | **Vitamin C (µg/g)** | | **Total Phenols (µg/g)** | | **Antioxidant (µg/g)** | | **Total soluble sugars (TSS) (µg/g)** | | **(C + X) (µg/mL)** | | **Chlorophyll a and b (µg/mL )** | | | |
| --- | --- | --- | --- | --- | --- | --- | --- | --- | --- | --- | --- | --- | --- | --- | --- | --- | --- |
|  |  |  |  |  |  |  |  |  |  |  |  |  |  | ***Chl a*** | | ***Chl b*** | |
|  |  | **(I)** | **(M)** | **(I)** | **(M)** | **(I)** | **(M)** | **(I)** | **(M)** | **(I)** | **(M)** | **(I)** | **M)** | **(I)** | **(M)** | **(I)** | **(M)** |
| 1 | AVR - 148 | Green | Yellow | 3340.52 | 6148.07 | 726.94 | 979.26 | 21.65 | 80.81 | 18696.78 | 2850.79 | 0.06 | 0.17 | 0.80 | 0.46 | 1.42 | 0.83 |
| 2 | F4-9(31A-5) | Green | Yellow | 2465.57 | 11523.07 | 157.26 | 1403.97 | 33.44 | 261.20 | 13988.55 | 12824.78 | 0.03 | 5.06 | 2.18 | 1.10 | 3.83 | 2.01 |
| 3 | F4-15(31-C-6) | Green | Yellow | 1626.22 | 7187.31 | 146.97 | 3095.11 | 35.88 | 219.54 | 20259.86 | 12218.45 | 0.05 | 0.37 | 1.82 | 0.85 | 3.21 | 0.95 |
| 4 | 33-A-5 | Green | Red | 2621.72 | 3267.12 | 571.07 | 2654.87 | 30.90 | 120.48 | 21079.30 | 6768.99 | 0.07 | 4.94 | 2.65 | 0.68 | 4.71 | 1.52 |
| 5 | F4-33A-1 | Green | Red | 2116.76 | 2667.23 | 154.11 | 2890.86 | 21.61 | 92.88 | 15968.32 | 8717.78 | 0.10 | 5.44 | 1.81 | 0.60 | 3.21 | 1.22 |
| 6 | F4-35B-1 | Green | Red | 3030.99 | 2607.35 | 1122.33 | 3836.50 | 20.78 | 78.09 | 33817.90 | 6732.56 | 0.05 | 4.94 | 1.50 | 0.38 | 2.65 | 0.99 |
| 7 | F4-37B-1 | Green | Red | 2212.75 | 8486.87 | 464.35 | 3174.26 | 40.63 | 384.22 | 33203.26 | 21737.56 | 0.04 | 5.46 | 2.32 | 0.42 | 4.05 | 0.97 |
| 8 | F4-36B-1 | Green | Red | 2310.78 | 4424.96 | 570.28 | 2649.45 | 27.54 | 121.53 | 4980.99 | 7476.54 | 0.14 | 6.93 | 2.45 | 0.86 | 4.27 | 1.11 |
| 9 | F4-37-B-2 | Green | Red | 1335.78 | 2541.20 | 488.08 | 2672.32 | 57.25 | 200.36 | 27278.97 | 8740.74 | 0.01 | 0.11 | 1.95 | 2.32 | 3.43 | 4.31 |
| 10 | F4-103B-1 | Green | Red | 808.66 | 1487.71 | 423.28 | 1373.10 | 37.00 | 114.20 | 31344.18 | 12123.04 | 0.05 | 2.92 | 2.41 | 5.23 | 4.83 | 9.12 |
| 11 | F4-103B-2 | Green | Red | 2072.64 | 2806.73 | 258.59 | 2111.39 | 9.77 | 149.26 | 18658.36 | 6504.00 | 0.06 | 0.12 | 1.75 | 0.61 | 3.08 | 1.12 |
| 12 | CPCT-144 | Green | Red | 2409.47 | 4288.01 | 222.75 | 1431.73 | 18.94 | 109.94 | 23190.64 | 7767.73 | 0.13 | 5.84 | 1.89 | 0.59 | 3.35 | 1.17 |
| 13 | Nishant | Green | Red | 2308.31 | 4621.52 | 456.83 | 2098.76 | 47.38 | 164.23 | 34501.66 | 11918.20 | 0.21 | 0.22 | 2.07 | 0.29 | 3.62 | 0.76 |
| 14 | KTC-152 | Green | Red | 2083.09 | 4786.99 | 369.90 | 1946.44 | 29.16 | 111.29 | 36979.09 | 12198.54 | 0.08 | 0.13 | 1.73 | 0.52 | 3.07 | 1.11 |
| 15 | California wonder | Green | Red | 2153.57 | 3849.88 | 398.96 | 1902.16 | 53.72 | 117.94 | 454.51 | 5007.92 | 0.38 | 7.95 | 2.22 | 0.41 | 3.91 | 0.79 |
| 16 | NBR - 16 | Green | Red | 2460.40 | 3269.70 | 699.02 | 3479.33 | 36.75 | 128.75 | 32368.07 | 12780.47 | 0.07 | 0.52 | 0.95 | 0.30 | 1.67 | 0.62 |
| 17 | NBR - 10 | Green | Red | 2837.50 | 5583.37 | 444.44 | 2687.41 | 27.65 | 103.80 | 38481.01 | 8928.14 | 0.05 | 3.03 | 2.00 | 7.16 | 3.51 | 12.55 |
| 18 | NBR - 19 | Green | Red | 2589.64 | 5320.90 | 289.26 | 1233.46 | 20.99 | 178.91 | 37488.28 | 10433.72 | 0.15 | 5.33 | 2.12 | 0.78 | 3.72 | 1.43 |
| 19 | NBR - 20 | Green | Red | 707.83 | 3634.25 | 375.12 | 5936.53 | 7.32 | 161.44 | 30256.66 | 14442.90 | 0.11 | 7.32 | 1.20 | 1.12 | 2.08 | 2.06 |
| 20 | NBR - 22 | Green | Red | 2439.19 | 11307.42 | 977.65 | 4068.46 | 34.80 | 85.38 | 38231.11 | 5734.56 | 0.11 | 7.34 | 2.07 | 0.45 | 3.62 | 0.81 |
| 21 | Pusa Capsicum | Green | Yellow | 1409.18 | 7126.97 | 168.50 | 1426.80 | 27.07 | 145.57 | 35014.57 | 8795.05 | 0.07 | 0.75 | 1.98 | 1.60 | 3.48 | 2.87 |
| 22 | Hybrid Indra | Green | Red | 397.78 | 3240.32 | 380.39 | 2483.96 | 31.37 | 137.95 | 43730.57 | 8324.78 | 0.04 | 5.59 | 3.40 | 2.19 | 5.99 | 3.96 |
| 23 | NBR-23 (White) | White | Orange | 934.17 | 12565.95 | 809.95 | 2930.53 | 63.67 | 377.41 | 42277.97 | 13958.14 | 0.17 | 3.15 | 0.46 | 0.42 | 0.81 | 0.73 |
| 24 | AVR-143 | Green | RED | 3617.21 | 5752.79 | 447.93 | 3781.14 | 31.41 | 187.92 | 23527.70 | 14549.95 | 0.08 | 4.19 | 0.46 | 5.33 | 0.81 | 9.34 |
| 25 | AVR - 154 | Green | Red | 427.69 | 2497.04 | 241.28 | 2717.17 | 31.92 | 105.16 | 36902.65 | 6612.33 | 0.39 | 8.20 | 0.61 | 1.20 | 1.11 | 2.18 |
| 26 | AVR - 141 | Green | Red | 2615.79 | 3791.20 | 523.27 | 3751.18 | 17.85 | 130.85 | 33282.40 | 8627.06 | 7.41 | 8.76 | 0.70 | 0.62 | 1.32 | 1.18 |
| 27 | NBR - 1 | Green | Red | 343.00 | 3693.30 | 247.81 | 2035.11 | 18.20 | 170.74 | 37651.29 | 4428.78 | 6.67 | 8.56 | 0.40 | 0.49 | 0.75 | 0.92 |
| 28 | NBR - 2 | Green | Red | 1721.87 | 3040.07 | 232.15 | 858.29 | 35.02 | 113.41 | 18362.50 | 5138.72 | 5.83 | 6.34 | 0.81 | 0.90 | 1.45 | 1.48 |
| 29 | LAXMI Hybrid check) | Green | Red | 8508.53 | 5092.30 | 3025.33 | 3795.83 | 175.50 | 255.98 | 19422.80 | 17482.20 | 0.65 | 0.47 | 0.81 | 0.82 | 0.83 | 0.89 |
| 30 | PURPLE | Purple | Red | 2688.23 | 5707.69 | 422.56 | 1929.30 | 29.46 | 153.31 | 43326.03 | 26622.23 | 8.77 | 1.00 | 3.39 | 1.05 | 1.73 | 1.02 |
| 31 | CHOCLATE | Green | Chocolate | 1721.87 | 8042.29 | 232.15 | 4857.88 | 35.02 | 390.39 | 18362.50 | 27483.07 | 4.50 | 5.19 | 3.50 | 0.91 | 1.65 | 1.01 |
| 32 | CHECK (KTPL-19) | Green | Red | 7064.05 | 7068.60 | 4308.12 | 6707.49 | 224.53 | 358.28 | 20679.50 | 30599.02 | 0.71 | 9.82 | 1.18 | 0.39 | 1.01 | 0.71 |
| 33 | AVR-152 | Green | Orange | 6979.81 | 3090.90 | 1730.84 | 2879.52 | 88.15 | 431.33 | 16505.95 | 28385.11 | 0.33 | 4.56 | 1.01 | 0.40 | 0.92 | 0.74 |
| 34 | AVR 127 | Green | Red | 5519.67 | 2404.85 | 4048.91 | 2870.31 | 202.71 | 289.69 | 23924.65 | 18271.48 | 0.23 | 1.14 | 0.59 | 0.45 | 0.71 | 0.74 |
| 35 | F5-32C-2 | Green | Yellow | 6437.36 | 6403.97 | 2808.90 | 2309.08 | 139.84 | 315.27 | 16468.56 | 14957.39 | 0.39 | 3.14 | 1.04 | 0.40 | 0.96 | 0.68 |
| 36 | F5-33A-2 | Green | Red | 8511.71 | 9379.15 | 2482.61 | 1917.62 | 177.07 | 187.81 | 16724.06 | 11098.29 | 0.27 | 0.49 | 0.86 | 1.46 | 0.87 | 1.19 |
| 37 | F5-32C-3 | Green | Yellow | 5912.13 | 7384.61 | 3138.25 | 5352.43 | 135.19 | 390.44 | 17624.89 | 30073.44 | 0.40 | 1.42 | 0.91 | 0.40 | 0.86 | 0.71 |
| 38 | F5-33A-5 | Green | Red | 6307.69 | 4769.23 | 2947.39 | 1489.79 | 205.28 | 286.49 | 26023.49 | 21811.46 | 0.45 | 2.85 | 1.21 | 0.52 | 0.81 | 0.79 |
| 39 | F5-33B-2 | Green | Red | 7427.92 | 7159.76 | 2206.26 | 3086.41 | 152.25 | 376.77 | 19059.53 | 19784.17 | 0.31 | 1.80 | 0.83 | 0.38 | 0.83 | 0.66 |
| 40 | F4-31-3 | Green | Yellow | 6784.59 | 3542.79 | 2830.47 | 2387.89 | 179.61 | 302.90 | 28058.04 | 22363.28 | 0.22 | 0.40 | 0.74 | 0.77 | 0.81 | 0.91 |
| 41 | F3-15-37 | Green | Yellow | 7745.11 | 7307.69 | 2640.66 | 1419.75 | 213.40 | 332.16 | 18374.10 | 22860.75 | 0.42 | 0.83 | 1.08 | 0.43 | 0.93 | 0.74 |
| 42 | F5-31D-2 | Green | Yellow | 6334.21 | 6854.11 | 1896.96 | 3045.67 | 123.74 | 321.71 | 15939.40 | 21013.49 | 0.72 | 0.97 | 0.76 | 0.41 | 0.81 | 0.71 |
| 43 | F4-31C-2 | Green | Yellow | 4347.49 | 4990.61 | 1878.10 | 1405.54 | 137.53 | 317.00 | 19256.56 | 16368.58 | 3.04 | 0.89 | 6.69 | 0.50 | 3.72 | 0.89 |
| 44 | F5-32A-1 | Green | Yellow | 5694.11 | 6133.33 | 2637.16 | 1547.84 | 192.54 | 296.67 | 16127.87 | 24689.24 | 0.33 | 1.45 | 0.79 | 0.43 | 0.78 | 0.68 |
| 45 | F5-31D-3 | Green | Yellow | 2745.56 | 7174.49 | 3673.43 | 3572.21 | 204.44 | 169.56 | 18596.78 | 12145.21 | 0.47 | 0.51 | 0.82 | 1.24 | 0.79 | 1.04 |
| 46 | F5-32C-1 | Green | Yellow | 7378.49 | 4666.51 | 2367.66 | 3379.20 | 121.88 | 288.45 | 15971.08 | 18462.00 | 1.23 | 0.56 | 0.98 | 0.53 | 0.91 | 0.81 |
| 47 | F5-33A-3 | Green | Yellow | 4292.22 | 7443.47 | 3160.04 | 2878.42 | 201.01 | 244.55 | 16955.05 | 10510.93 | 0.44 | 0.54 | 1.07 | 1.27 | 1.01 | 1.15 |
| 48 | F5-32A-2 | Green | Yellow | 4472.27 | 8630.76 | 3270.37 | 2266.14 | 184.11 | 167.69 | 20023.23 | 12416.00 | 0.39 | 1.57 | 1.06 | 0.38 | 0.92 | 0.66 |
| 49 | AVR 151 | Green | Yellow | 4616.63 | 3310.61 | 2286.89 | 2271.78 | 128.15 | 163.29 | 12108.66 | 9999.52 | 0.34 | 1.14 | 1.01 | 0.39 | 0.88 | 0.68 |

C+X- Carotenoids & Xanthophyll

**Supplementary Table 2.** Mean of anthocyanin µg/g during different growth stages of capsicum

| **Genotype** | **Anthocyanin µg/g (IMM)** | **Anthocyanin (µg/g) (M)** |
| --- | --- | --- |
| Hybrid Laxmi | -1.669888476 | -1.06873 |
| PURPLE | 15.49656506 | -0.40077 |
| CHOCLATE | -1.870275093 | 1.135524 |
| KTPL-19 (CHECK) | 14.36104089 | -0.60116 |
| AVR-152 | -1.469501859 | -0.73475 |
| AVR 127 | -0.601159851 | 0.935138 |
| F5-32C-2 | -0.534364312 | 0.133591 |
| F5-33A-2 | 1.335910781 | 0.200387 |
| F5-32C-3 | -1.269115242 | 0.133591 |
| F5-33A-5 | -0.400773234 | -0.86834 |
| F5-33B-2 | -0.133591078 | -0.73475 |
| F4-31-3 | -0.267182156 | -1.40271 |
| F3-15-37 | 0.267182156 | -0.60116 |
| F5-31D-2 | 0.66795539 | -0.46757 |
| F4-31C-2 | -0.133591078 | -0.46757 |
| F5-32A-1 | -0.66795539 | -0.53436 |
| F5-31D-3 | -1.469501859 | -0.86834 |
| F5-32C-1 | -0.267182156 | -0.60116 |
| F5-33A-3 | 3.47336803 | -0.46757 |
| F5-32A-2 | -0.601159851 | 2.337844 |
| AVR 151 | 2.605026022 | 0.200387 |
| NBR-22 | 4.007732342 | -0.13359 |
| F4-37B-1 | 2.471434944 | -7.28071 |
| F3-13-2 (White) | -2.271048327 | 6.278781 |
| F4-31A-5 | -0.801546468 | 1.736684 |
| F4-31C-6 | -2.7386171 | 0.868342 |
| AVR - 148 | 8.149055762 | 4.608892 |
| 33-A-5 | -9.885739777 | 1.402706 |
| F4-33A-1 | 4.542096654 | -2.33784 |
| F4-35B-1 | 2.939003717 | -0.80155 |
| F4-36B-1 | -3.940936803 | 4.074528 |
| F4-37-B-2 | 1.001933086 | 2.53823 |
| F4-103B-1 | 9.217784387 | 5.811212 |
| F4-103B-2 | 7.61469145 | 1.736684 |
| CPCT-144 | -5.076460967 | -0.46757 |
| Nishant | -19.5042974 | -1.00193 |
| KTC-152 | 2.07066171 | -1.5363 |
| California Wonder | 7.347509294 | 4.876074 |
| NBR - 16 | -3.206185874 | 6.679554 |
| NBR - 10 | 5.143256506 | 4.809279 |
| NBR - 19 | -0.133591078 | 2.671822 |
| NBR - 20 | 8.750215613 | -5.9448 |
| Pusa Capsicum | -3.874141264 | -2.27105 |
| Hybrid Indra | -0.534364312 | -2.60503 |
| AVR - 154 | 5.677620818 | 3.005799 |
| AVR - 141 | -4.007732342 | -5.67762 |
| NBR - 1 | -1.603092937 | -2.87221 |
| NBR - 2 | -5.410438662 | 0.534364 |
| Standard deviation | 5.688 | 2.904 |
