# Supplementary material for: Phytochemical characterization and correlation analysis of nutritional value in sweet pepper (Capsicum annuum L.) genotypes at various growth stages
Source: Front Plant Sci. 2026 Feb 11;16:1719537. doi: 10.3389/fpls.2025.1719537 (PMC12932425; doi:10.3389/fpls.2025.1719537)
Supplement: Supplementary Figure 1 — Principal Component Analysis (PCA) biplot showing the distribution of genotypes based on seven biochemical traits at immature and mature growth stages. [file Supplementaryfile1.docx]

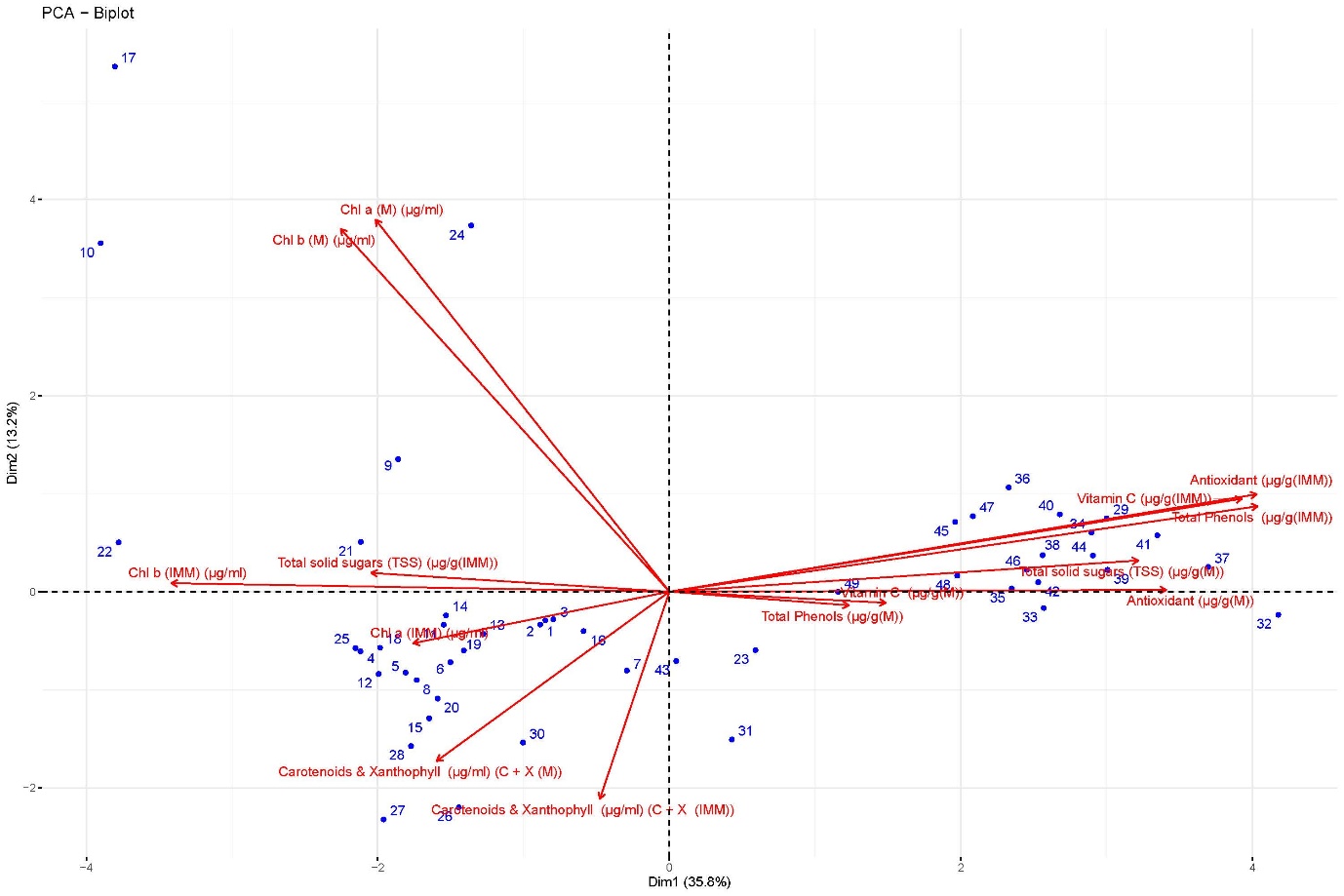


**Supplementary Figure S1.** A principal component analysis (PCA) was conducted to explore the relationships among genotypes based on biochemical traits.


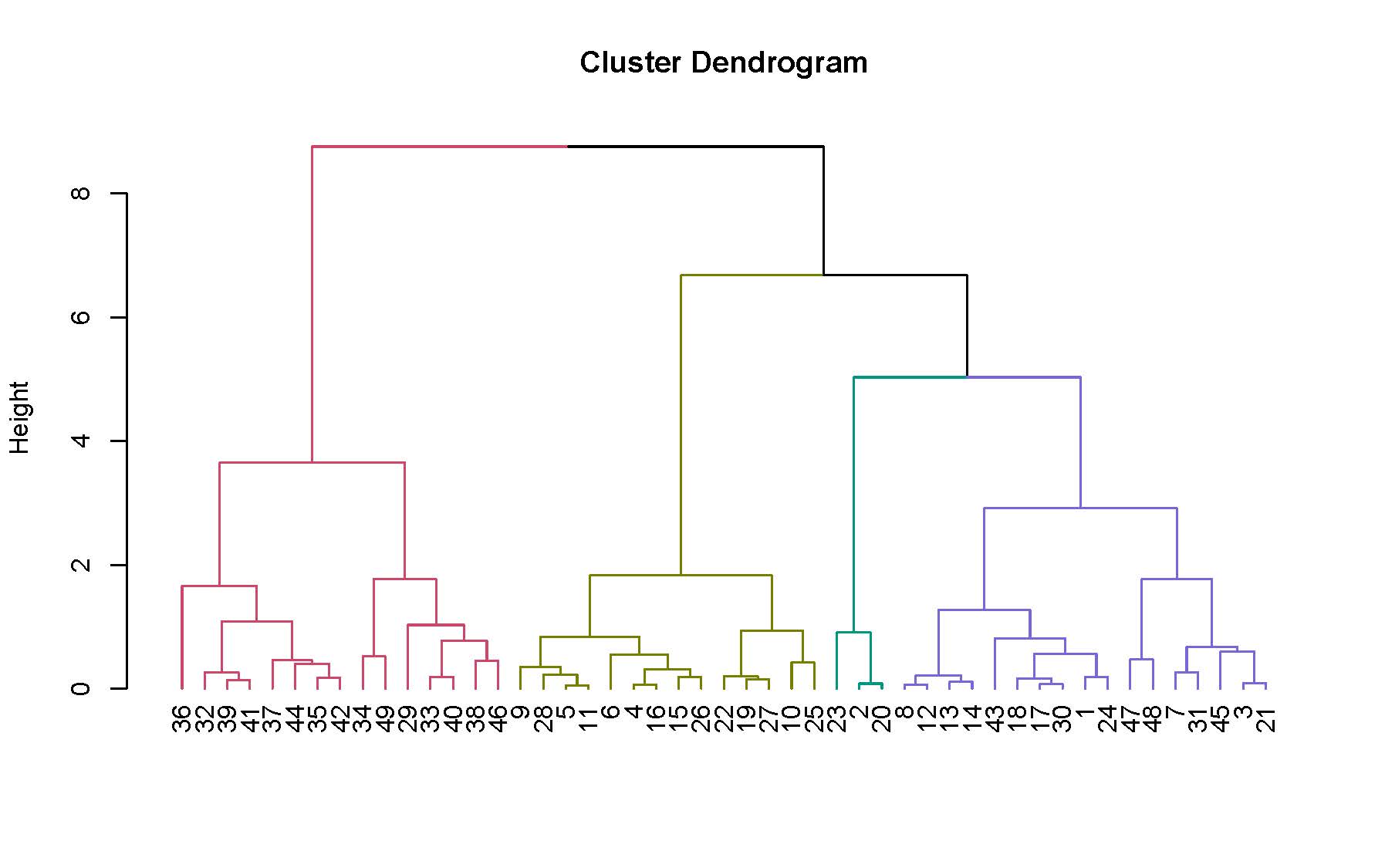


**Supplementary Figure S2.** Hierarchical cluster analysis to examine the genetic relationships among the tested genotypes.
